# Development and In Vitro Evaluation of 5-Fluorouracil-Eluting Stents for the Treatment of Colorectal Cancer and Cancer-Related Obstruction

**DOI:** 10.3390/pharmaceutics13010017

**Published:** 2020-12-24

**Authors:** Mohammad Arafat, Paris Fouladian, Anthony Wignall, Yunmei Song, Ankit Parikh, Hugo Albrecht, Clive A. Prestidge, Sanjay Garg, Anton Blencowe

**Affiliations:** 1 Pharmaceutical Innovation and Development (PIDG) Group, Clinical and Health Sciences, University of South Australia (UniSA), Adelaide, SA 5000, Australia; mohammad.arafat@mymail.unisa.edu.au (M.A.); paris.fouladian@mymail.unisa.edu.au (P.F.); May.Song@unisa.edu.au (Y.S.); Ankit.Parikh@unisa.edu.au (A.P.); 2 ARC Centre of Excellence in Convergent Bio-Nano Science and Technology, Clinical and Health Sciences, University of South Australia (UniSA), Adelaide, SA 5000, Australia; anthony.wignall@unisa.edu.au (A.W.); Clive.Prestidge@unisa.edu.au (C.A.P.); 3 Drug Discovery and Development Group, Clinical and Health Sciences, University of South Australia (UniSA), Adelaide, SA 5000, Australia; Hugo.Albrecht@unisa.edu.au; 4 Applied Chemistry and Translational Biomaterials (ACTB) Group, Clinical and Health Sciences, University of South Australia (UniSA), Adelaide, SA 5000, Australia

**Keywords:** 5-fluorouracil, drug-eluting stent, gastrointestinal cancer, dip-coating, localised drug delivery

## Abstract

Self-expanding metal stents (SEMSs) are currently the gold standard for the localised management of malignant gastrointestinal (GI) stenosis and/or obstructions. Despite encouraging clinical success, in-stent restenosis caused by tumour growth is a significant challenge. Incorporating chemotherapeutic drugs into GI stents is an emerging strategy to provide localised and sustained release of drugs to intestinal malignant tissues to prevent tumour growth. Therefore, the aim of this work was to develop and evaluate a local GI stent-based delivery system that provides a controlled release of 5-fluorouracil (5FU) over a course of several weeks to months, for the treatment of colorectal cancer and cancer-related stenosis/obstructions. The 5FU-loaded GI stents were fabricated via sequential dip-coating of commercial GI stents with a drug-loaded polyurethane (PU) basecoat and a drug-free poly(ethylene-*co*-vinyl acetate) (PEVA) topcoat. For comparison, two types of commercial stents were investigated, including bare and silicone (Si) membrane-covered stents. The physicochemical properties of the 5FU-loaded stents were evaluated using photoacoustic Fourier-transform infrared (PA-FTIR) spectroscopy, X-ray diffraction (XRD), X-ray photoelectron spectroscopy (XPS), scanning electron microscopy (SEM), and thermal analysis. In vitro release studies in biological medium revealed that the 5FU-loaded stents provided a sustained release of drug over the period studied (18 d), and cell viability, cell cycle distribution and apoptosis assays showed that the released 5FU had comparable anticancer activity against human colon cancer cells (HCT-116) to pure 5FU. This study demonstrates that dip-coating is a facile and reliable approach for fabricating drug-eluting stents (DESs) that are promising candidates for the treatment of GI obstructions and/or restenosis.

## 1. Introduction

Colorectal cancer (CRC) remains the second leading cause of cancer deaths and the third most commonly diagnosed cancer globally. More than 1.8 million new CRC cases and 881,000 deaths from CRC were estimated worldwide in 2018. Approximately 8 to 29% of patients diagnosed with CRC initially manifest with acute colonic obstruction and/or stenosis. Among them, more than 70% of patients already have advanced CRC at diagnosis, and only half of these patients are suitable candidates for curative one-stage surgery [1,2]. Depending on the anatomical location and extent of the obstruction (partial or complete), many of these patients encounter difficulties in ingesting food and need urgent medical interventions to improve or restore their colonic luminal patency and digestive functions [3,4]. In this clinical setting, an enteral stent is inserted as a nonsurgical alternative in CRC patients, mainly as a palliative treatment of unresectable malignant colonic strictures or as a bridge to elective surgery in patients with potentially curable malignant CRC obstruction [2,3,5,6,7,8].

While colonic stenting has been performed with high technical and clinical success rates in the management and palliation of obstructive metastatic CRC for the past 20 years [1,2,3,4,6,7,9], conventional colonic stents commonly face the problem of occlusion due to growth of malignant tumour cells [1,4,7,9,10]. All commercial GI stents available to date act as endoluminal scaffolds and only provide mechanical palliation of the obstruction without antitumour activity [11,12]. This major limitation of traditional GI stents often results in a reduction in the effective duration of the stenting treatment, and so further reinterventions using another stent or other palliation treatment are often required [5,8,9].

To address the limitations associated with currently used colonic stenting, there has been increasing interest in drug-eluting GI stents [10,11,12,13,14,15,16,17,18,19,20,21,22,23], following the great clinical success of drug-stent combination products in the cardiovascular field [24]. GI stents have been investigated in combination with contemporary chemotherapeutic drugs to act both as a normal GI stenting device and provide a drug reservoir. Thus, GI DESs are primarily intended to deliver the chemotherapeutic drug directly at the stent implantation site in a controlled fashion, with the aim of maximising the drug bioavailability within local GI tumour tissue and minimising systemic toxicities. Therapeutics released from DESs over a course of weeks to months are more likely to result in high local concentrations in the immediate vicinity of tumours [10,14,18,21,22], and therefore, DESs have great potential to efficiently suppress the growth of tumour cells over the stent compared to traditional administration of the stent and the drug separately [10,12,14,17,18,19,21,22,23,25,26].

DESs contain three primary components: the stent platform, drug substance and a polymer coating/carrier system [27,28]. Currently, a number of US Food and Drug Administration (FDA)-approved anticancer drugs are used routinely in the treatment of GI cancers, including 5FU, leucovorin, capecitabine, irinotecan, oxaliplatin, and bevacizumab [29,30]. Although it is widely acknowledged that the total drug dose to be loaded in a DES is significantly lower than that required for conventional oral or systemic applications, the selection of the most appropriate chemotherapeutic drug or drug combinations has not been established [29,31]. Nevertheless, among the clinically available anticancer drugs, 5FU is one of the most potent and effective drugs against many solid tumours and has been used as the mainstay treatment of many different cancer types for over 40 years, including GI tract cancers. 5FU is mainly given intravenously as a result of its low oral absorption, which can cause unacceptable neural, haematologic, cardiac, GI and dermatologic side effects and/or toxicities. Additionally, a significant portion (>85%) of the systemically administered dose of 5FU is rapidly catabolised into inactive metabolites, leading to a short in vivo circulation time (biological half-life: 10 to 20 min) and low drug availability near the site of action [10,12,14,29,32]. Thus, 5FU is a good candidate for localised, controlled and low-dose delivery from DESs.

Another important component of DESs is the drug-carrying polymer, which must have adequate capacity to encapsulate the required amount of drug and release the drug in a controlled fashion over a particular period of time [28,33]. While bioresorbable polymers have been investigated for non-vascular DESs, biodegradation of the coating can result in stent migration, stricture recurrence, or early stent blockage due to malignant tumour growth [34]. Commonly used biostable polymers for DES fabrication include PUs, silicones and PEVA [27,34,35]. In particular, PUs exhibit good shape-memory and are suitable for incorporating a wide variety of drugs [34]. Nevertheless, the high solubility of 5FU can result in a rapid release profile, and therefore, a drug-free PEVA outer layer has been used to slow the release kinetics [36]. The non-biodegradable thermoplastic PEVA is a random copolymer composed of two different monomer units; semi-crystalline segments of polyethylene (PE) and amorphous segments of poly(vinyl acetate) (PVA). The physical and mechanical properties of PEVA vary dramatically with the change in its vinyl acetate (VA) content, as the polymer crystallinity also changes. With the increase of the VA content, the crystallinity of PEVA decreases but the polarity increases [37].

While a number of preclinical and clinical studies have been carried out to investigate the safety and effectiveness of GI DESs [13,15,16,17,18,19,20,22,23], only a few have addressed stent-based localised delivery of 5FU [10,12,14,21,38]. Liu et al. and Guo et al., evaluated the in vivo efficacy of 5FU-loaded oesophageal DESs on porcine and rabbit models respectively, and revealed that 5FU concentrations in the oesophageal tissue were significantly higher compared with peripheral organs, supporting the case for localized delivery and reduced systemic toxicity [14,21]. In addition, Li et al. developed a series of 5FU-loaded biodegradable stents and showed in in vivo mice studies that higher 5FU loadings resulted in superior antitumour effects [12].

Various types of polymer coating techniques (e.g., dip-coating, spray coating, electrospinning, and hot-melt coating) have been employed to fabricate DESs, although dip-coating is commonly regarded as the simplest method [11,13,15,20,22,39,40]. Nevertheless, to date there are no examples of dip-coated 5FU-loaded stents, which highlights the challenges of preparing appropriate 5FU-polymer formulations suitable for dip-coating. Therefore, in this study we aimed to demonstrate the potential advantages of dip-coating to reproducibly and economically manufacture DESs for the controlled and prolonged delivery of 5FU for the treatment of CRC. Furthermore, we conducted the first comparative study to evaluate the in vitro physicochemical and biological properties of anticancer drug-loaded stents fabricated with the same drug-polymer formulation but using two different types of GI stents.

## 2. Materials and Methods

### 2.1. Materials

Clinical, non-vascular self-expanding nitinol (nickel-titanium alloy) stents with Si membrane-coverings (Niti-S™ S-type biliary stents, diameter of 1 cm) and uncovered (bare) stents (Niti-S™ D-type pyloric/duodenal/enteral colonic stents, diameter of 2.2 cm) were kindly provided by Taewoong Medical Co., Ltd (Gimpo-si, Gyeonggi-do, South Korea). Single side polished N-type silicon wafers (resistivity 100 to 3000 Ω.cm and thickness 0.50 mm) were purchased from Sigma-Aldrich Pty Ltd (a subsidiary of Merck; North Ryde BC, NSW, Australia), diamond cut into small pieces, cleaned/oxidised with piranha solution (a 3:1 mixture of concentrated sulfuric acid (98%) and hydrogen peroxide aqueous solution (30% *w/v*), rinsed thoroughly with Milli-Q water and air-dried before use. Glass microscope slides (length 76 mm, width 26 mm, thickness 1 mm) were purchased from Paul Marienfeld GmbH & Co. KG (Am Wöllerspfad, Lauda-königshofen, Germany). 5FU was purchased from Dayang chem (Hangzhou) Co., Ltd. (Hangzhou, Zhejiang, China). ChronoFlex AL, a medical grade aliphatic polycarbonate-based thermoplastic urethane was kindly gifted by AdvanSource Biomaterials Corporation (Wilmington, MA, USA; Supplementary Information (SI), Figures S1–S3). PEVA (VA 40 wt%) (SI, Figure S3), *N,N*-dimethylformamide (DMF, HPLC grade), RPMI-1640 medium, trypsin, ethylenediaminetetraacetic acid (EDTA), and bovine pancreatic ribonuclease (RNase A) solution were purchased from Sigma-Aldrich Pty Ltd. Sulfuric acid (ACS Reagent grade), hydrogen peroxide 35% *w/w* (aqueous solution, laboratory reagent grade), tetrahydrofuran (THF, ACS reagent grade), and dimethyl sulfoxide (DMSO, ACS reagent grade) were purchased from Chem-Supply Pty Ltd (Gillman, SA, Australia). Dichloromethane (DCM, HPLC grade) and acetonitrile (ACN, HPLC grade) were purchased from Merck KGaA (Darmstadt, Germany). HCT-116 cell line was obtained from the cell bank at the Centre for Drug Discovery and Development, University of South Australia (Adelaide, SA, Australia). Fetal bovine serum (FBS) and fluorescein isothiocyanate (FITC) Annexin V apoptosis detection kit were procured from Life Technologies Australia Pty Ltd (Mulgrave, Victoria, Australia) and BD Biosciences-Australia (North Ryde, NSW, Australia), respectively. All reagents were used as received unless otherwise stated.

### 2.2. Methods

#### Fabrication of 5FU-Loaded Drug-Eluting GI Stents and Substrates

DESs were prepared via the sequential dip-coating of commercial stents with a 5FU-loaded PU basecoat and a drug-free PEVA topcoat (outermost) (SI, Table S1). Initially, PU polymer beads (35 g) were dissolved in THF (173 mL) at 60 °C with agitation to afford a 20.3% *w/v* solution, and the temperature was reduced to 40 °C for 30 min. Separately, 5FU (2.41 g) was dissolved in DMF (27.4 mL) with sonication (Model 5510E-DTH, Branson Ultrasonics Corporation, Danbury, CT, USA) to afford a 8.81% *w/v* solution. The 5FU solution was then added dropwise with sonication to the PU solution and sonicated for a further 1 h. The resulting solution was maintained at 40 °C and used immediately to coat Si-covered and bare nitinol stents via dip-coating (refer to SI, Table S2 for dip-coating parameters) with a desktop dip-coater (Model TL0.01, MTI Corporation, Richmond, CA, USA) (SI, Figure S4). The resulting base-coated stents were kept in an oven at 60 °C for 36 h and then weighed to determine the mass of the basecoat, which consisted of 6.5% *w/w* of 5FU. The drug-free topcoat solution was prepared by dissolving PEVA (52 g) in DCM (200 mL) with sonication over 4 h. The PEVA solution was dip-coated onto the stents, air dried for 24 h, and then weighed to determine the mass of the topcoat. Drug-free (blank) control stents, with and without PEVA topcoat, were fabricated identically and under the same experimental conditions as described above, but without the addition of drug.

To facilitate physicochemical characterisation of the drug-polymer coatings, glass microscope slides and silicon wafers were also dip-coated and dried using the same coating formulations, dip-coating machine, and process parameters described previously for the stents. Furthermore, for the purposes of solid-state characterisation of the drug-incorporated PU basecoat, 5FU-loaded PU films and drug-free (blank) PU films were prepared via film-casting using the same basecoat formulation as used for dip-coating.

### 2.3. Characterisation

To facilitate optimal characterization of the films and coated stents, specific samples were used for different analytical techniques (SI, Table S3).

#### 2.3.1. Photoacoustic Fourier-Transform Infrared (PA-FTIR) Spectroscopy

PA-FTIR spectroscopy was performed on silicon wafers dip-coated with single (basecoat) or double (basecoat and topcoat) polymer layers, and pure 5FU powder. PA-FTIR spectra were recorded from 400 to 4000 cm^−1^ using a Magna-IR spectrometer (Model 750, Nicolet Instrument Corporation, Madison, WI, USA) equipped with an MTEC Model 300 photoacoustic (PA) cell. For each sample spectrum 128 scans were averaged at a resolution of 8 cm^−1^. A carbon black reference sample was used to record the background spectrum prior to sample analysis.

#### 2.3.2. X-Ray Diffraction (XRD)

XRD was carried out on solvent cast 5FU-loaded and blank PU films, and pure 5FU powder, using a Panalytical Empyrean X-ray diffractometer (Malvern Panalytical Ltd, Malvern, Worcestershire, UK) operating at 40 mA and 40 kV with Cu Kα radiation (λ = 1.541874 Å). The analysis was conducted at a 6° take-off angle and the samples were scanned over the 2*θ* range of 5° to 40° with a step size and step time of 0.026° and 179.52 s, respectively. The corresponding interlayer distances or *d*-spacing values were calculated from the XRD experimental parameters using Bragg’s equation [41,42,43].

#### 2.3.3. X-Ray Photoelectron Spectroscopy (XPS)

XPS was performed on polymer films (10 × 10 mm^2^) that were cut and peeled off glass slides that had been dip-coated with the basecoat or both basecoat and topcoat layers. Spectra were recorded at 4 × 10^−8^ Torr using an AXIS Ultra spectrometer (Kratos Analytical Ltd, Wharfside, Manchester, UK) equipped with a delay-line detector (DLD) and monochromatic Al Kα x-ray source operating at 225 W with a characteristic energy of 1486.69 eV. The electron take-off angle was normal with respect to the surface of each sample and survey spectra were collected with an analysis area (iris aperture) of 0.30 × 0.70 mm^2^, an analysis depth of ~15 nm, a pass energy of 160 eV, and a dwell time of 55 ms. The charge neutralizer system was used during analysis of each samples. Peak position references were taken from the National Institute of Standards and Technology (NIST) X-ray Photoelectron Spectroscopy Database [44], the Handbook of X-ray Photoelectron Spectroscopy [45] and the XPS of Polymers Database [46]. All spectra have been charge-corrected with respect to the adventitious carbon (1s spectrum) fixed at 285 eV and spectral data were interpreted using CasaXPS software, version 2.3.22PR1.0 (Casa Software Ltd., Teignmouth, Devon, UK).

#### 2.3.4. Scanning Electron Microscopy (SEM)

For topography images of the abluminal (outer) surfaces of the samples, coated stents were cut into pieces with scissors, placed horizontally on double sided adhesive carbon tape fixed on an aluminum SEM stub, and then platinum (Pt)-sputtered (thickness ~5 nm) using an automatic sputter coater (Agar Scientific Ltd, Stansted, Essex, UK). Images were acquired using a Zeiss Merlin field emission gun (FEG) scanning electron microscope (Jena, Thuringia, Germany) operating at an accelerating voltage of 2 kV. For cross-sectional imaging, the stent pieces were positioned vertically on a specially designed SEM stub and secured with a screw. Cross-sectional SEM images were recorded using a Quanta 450 FE environmental scanning electron microscope (FEI Company, Hillsboro, Oregon, USA) operating at an accelerating voltage of 2 kV and at 2.5 spot size. For each sample, film thickness measurements were recorded using ImageJ (Version 1.52a) image analysis software program (National Institutes of Health, Bethesda, MD, USA) from the cross-sectional SEM images.

#### 2.3.5. Thermal Analysis

Differential scanning calorimetry (DSC) and thermogravimetric analysis (TGA) were carried out on solvent cast 5FU-loaded and blank PU films, and pure 5FU drug using a Discovery DSC 2920 (TA Instruments, New Castle, DE, USA) and Discovery TGA 550 (TA Instruments), respectively. For DSC, accurately weighed films or 5FU (~2 mg) were placed in aluminum pans and hermetically sealed, and then measurements were performed at a heating rate of 10 °C/min between 20 and 300 °C under a nitrogen atmosphere. For TGA, ~7.6 mg of sample was placed in a platinum pan and then heated under a nitrogen gas flow at a rate of 10 °C/min up to 500 °C.

#### 2.3.6. HPLC Determination of 5FU

5FU was quantified using the reversed-phase HPLC method described in the United States Pharmacopeia-National Formulary (USP-NF) [47]. Briefly, a Prominence HPLC system (Shimadzu Corporation, Kyoto, Japan) equipped with a SPD-M20A photodiode array UV–Vis detector, was fitted with a octadecylsilane (C18) column (length: 250 mm, internal diameter: 4.60 mm, particle size: 5 µm; Phenomenex Australia Pty Ltd. (Lane cove west, NSW, Australia)) at a wavelength of 265 nm. The mobile phase was composed of acetonitrile (CH_3_CN) and monobasic potassium phosphate (kH_2_PO_4_) buffer (6.80 g/L, pH: 5.70 ± 0.10) at a 5:95 ratio, and a 20 µL of sample was injected at a flow rate of 1 mL/min for each analysis.

#### 2.3.7. In Vitro Drug Release

The full-length Si-covered and bare stents (both 5FU-loaded and drug-free blank controls) were cut into small pieces weighing 100.90 ± 0.00 and 104.97 ± 0.05 mg, respectively, with a sharp stainless steel (SS) scissor. The bilayer coated Si-covered and bare stent pieces (*n* = 3 for each) were placed in 5 mL sterile tubes with 4.0 and 3.2 mL of 10% *v/v* FBS-supplemented sterile RPMI-1640 medium, respectively. The tubes were then sealed and transferred to a horizontal shaker (20 mm orbital diameter, 175 rpm) at 37 °C. The entire release medium from each tube was collected daily and subsequently replaced with fresh release medium (4.0 mL or 3.2 mL). The concentration of 5FU in the collected release medium was measured using HPLC (*vide supra*).

### 2.4. In Vitro Anticancer Activity of 5FU-Loaded GI Stents

To assess the anticancer activity of the 5FU-loaded bilayer coated stents, the 10% FBS-supplemented RPMI-1640 medium collected periodically from drug release studies was used for in vitro cell studies. Release medium collected on day 1, 7 and 14 was filtered (sterile 0.20 µm cellulose acetate (CA) filters) in a Thermo Scientific Safe 2020 class II laminar flow cabinet (Thermo Electron LED GmbH, Robert-Bosch-Strasse 1, Langenselbold, Germany) and used directly for cytotoxicity experiments, whereas for cell cycle and apoptosis analysis the release medium was diluted 5-fold with complete RPMI-1640 medium. As a positive control, a series of 5FU drug solutions at different concentrations were prepared in complete RPMI-1640 medium.

#### 2.4.1. Cell Culture and Maintenance

HCT-116 cells from passage 15 to 25 were grown in sterile T-25 rectangular canted neck cell culture flasks (25 cm^2^ surface area, 50 mL capacity) using complete RPMI-1640 medium supplemented with 10% *v/v* heat-inactivated FBS. Cell cultures were incubated at 37 °C and 5% CO_2_ / 95% air (Heracell 150i CO_2_ incubator, Thermo Scientific, Scoresby, VIC, Australia) with medium changes every two days. The cells were passaged at a 1:10 split ratio once cell density reached 80% confluency by treating with 0.25% trypsin and 0.02% EDTA for 5 min after washing with sterile PBS.

#### 2.4.2. Cytotoxicity Study

Cytotoxicity was assessed using the colorimetric (3-(4,5-dimethyl-2-thiazolyl)-2,5-diphenyl-tetrazolium bromide) MTT cell viability assay [12,17,18,19,48,49,50,51]. HCT-116 cells were seeded in sterile 96-well tissue culture plates at a density of 5 × 10^4^ cells/well and incubated for 24 h at 37 °C, 5% CO_2_. The test samples (100 µL of stent-release media or 5FU) were added into individual wells and incubated for 72 h, along with corresponding drug-free (blank) stent and untreated media controls. The medium was carefully removed from each well and the cells were washed with sterile PBS (pH 7.40, 100 µL). MTT reagent solution (100 μL, 5 mg/mL in sterile PBS, pH 7.40) was added to the treated cells and they were incubated at room temperature, in the dark for 4 h. The MTT reagent solution was removed, DMSO (100 μL) was added to each well and the plates were placed on an orbital shaker for 10 min. The absorbances of the DMSO aliquots were measured at 540 nm using a VICTOR3 multilabel counter (model 1420-012, WALLAC Oy, Turku, Finland/PerkinElmer, Singapore). The results were expressed as the percentage viability of cells treated with 5FU, stent-release media or drug-free stent media compared to untreated (control) cells, by calculating the average of all the replicate measurements with standard error. The concentration of 5FU that induced 50% of the cell viability or the maximum growth inhibition (IC_50_) was calculated using the GraphPad Prism version 7.03 software (GraphPad Software, Inc., San Diego, CA, USA) with a four-parameter logistic (4PL) curve-fit. In addition, HCT-116 cells were also exposed to the 10% FBS-supplemented RPMI-1640 medium alone as a negative control treatment (only for MTT assay).

#### 2.4.3. Cell Cycle Analysis and Detection of Apoptosis by Flow Cytometry

For cell cycle analysis, HCT-116 cells were seeded at a concentration of 1 × 10^6^ cells/well in 6-well plates and incubated for 16 h at 37 °C, 5% CO_2_. The cells were treated separately in duplicate with 1 mL from each of the test and control samples and incubated for 24 and 48 h. The supernatant was collected, along with the remining cellular monolayers that were harvested by trypsinisation, gently washed once and resuspended in PBS (pH 7.40). Cells were then fixed in 500 μL of ice-cold 70% (*v/v*) ethanol, mildly vortexed to ensure the collection of singlets and kept at 4 °C for 15 min before being pelleted and resuspended in PBS. After that, the cells were again pelleted and resuspended in dark environment with 200 μL of staining solution (50 µg/ml propidium iodide (PI), 100 µg/mL RNase A, and 500 µg/mL Triton X-100) in PBS and incubated at 37 °C for additional 1.5 h. Fluorescence intensity of at least 10,000 stained cells per sample was measured on a BD LSRFortessa™ cell analyser (BD Biosciences, San Jose, CA, USA), while the data obtained were processed/analysed using their companion software FCS express v6 [12,18,48,49].

For cell apoptosis analysis, the FITC Annexin V apoptosis detection kit (BD Biosciences) was used. HCT-116 cells were seeded at a density of 1 × 10^5^ cells/well in 10% *v/v* FBS-supplemented RPMI-1640 cell growth medium in 48-well plates, and incubated for 24 h at 37 °C, 5% CO_2_. The test samples (100 µL of stent-release media or 5FU) were added into individual wells, supplemented with additional FBS (10 μL) and incubated at 37 °C for 24 h and 48 h. At each time point, cells were collected, gently washed with PBS (pH 7.40) by centrifugation and diluted to 1 × 10^5^ cells/mL. Thereafter, the collected cell pellets were resuspended in ice-cold Annexin V binding buffer (100 μL) and FITC Annexin V and PI staining solutions (3 μL of each) were added to each cell suspension with mild vortexing. The cell suspensions were incubated at room temperature in the dark for 15 min and Annexin V binding buffer (200 μL) was added to each sample prior to flow cytometric analysis within an hour of staining. All experiments were performed at least in duplicate and the data were analysed using the FCS express v6 software from BD Biosciences [12,17,18,19,48,49].

## 3. Results and Discussion

### 3.1. Preparation of Dip-Coated 5FU-Loaded GI Stents

**Figure 1:**
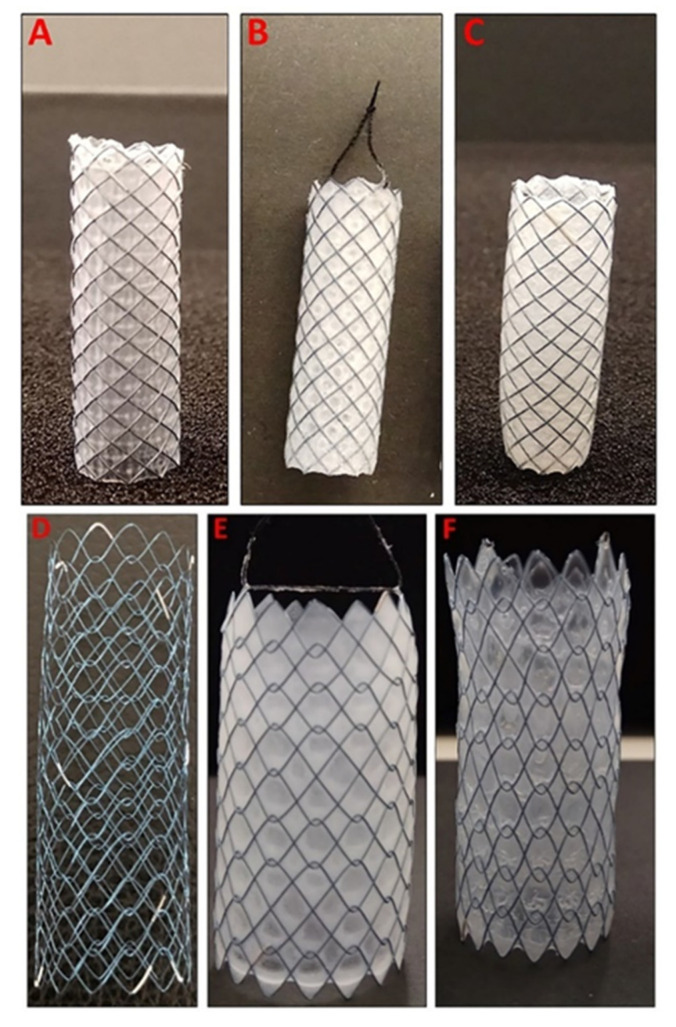
(**A**) Silicone membrane-covered self-expanding nitinol stent (fixed cells with braided structure), (**B**) Si-PU_5FU_ stent and (**C**) Si-PU_5FU_-PEVA stent. (**D**) Uncovered (bare) self-expanding nitinol stent (unfixed cells with weaving structure), (**E**) B-PU_5FU_ stent and (**F**) B-PU_5FU_-PEVA stent.

5FU-loaded stents were fabricated via sequential dip-coating of commercially available GI nitinol stents with a drug-loaded PU basecoat solution followed by a drug-free PEVA topcoat solution. While the basecoat acts as the drug reservoir, the topcoat was intended to mediate the diffusion and release of the highly hydrophilic 5FU. Two types of nitinol stents (bare and silicone membrane-covered) were employed (Figure 1) to assess the applicability of the coating process to different types of SEMSs, and compare potential differences in performance. The coated stents were designated as B-PU_5FU_-PEVA and Si-PU_5FU_-PEVA for bare and silicone stents coated with 5FU-loaded PU then PEVA, respectively.

While double or multiple coated stents have been prepared previously [36,52,53,54], the homogeneous incorporation of hydrophilic drugs within a hydrophobic polymer matrix can be challenging, particularly using dip-coating. Therefore, we initially optimised a coating formulation that would provide high loadings of 5FU within a PU matrix. For the hydrophobic PU matrix, the non-biodegradable ChronoFlex AL was selected due to its good biocompatibility, and thermal and mechanical stability [24,55]. A homogeneous coating solution of the PU and 5FU was prepared using a binary solvent (THF:DMF 6.3:1, *v/v*), which was then used to dip-coat the stents, resulting in B-PU_5FU_ and Si-PU_5FU_ stents and provided a PU basecoat with a high 5FU-loading of 6.5% *w/w* (Figure 1).

Subsequently, a PEVA topcoat was applied as an elution-controlling layer and to avoid the premature burst release of 5FU from the basecoat [36,53,54,56]. In this study, PEVA with a VA content of 40 wt% was used, but as demonstrated previously the drug release rate can be modulated by changing the VA content or thickness of PEVA coatings [10,14,21,36,57,58]. The PEVA topcoat was dip-coated onto the stents using a 26% *w/v* DCM solution to afford B-PU_5FU_-PEVA and Si-PU_5FU_-PEVA stents (Figure 1). DCM was selected as it is a good solvent for PEVA, a non-solvent for 5FU, and to minimise swelling of the PU basecoat; the rate of swelling of the PU in DCM was found to be negligible compared to the time required for the dip-coating process. A 26% *w/v* PEVA solution was employed as lower concentrations resulted in low coating thicknesses and higher concentrations resulted in non-uniform coatings due to the high solution viscosities.

Using the dip-coating process described above and optimized dip-coating parameters (SI, Table S2), B-PU_5FU_-PEVA and Si-PU_5FU_-PEVA stents (*n* = 10 for each) were prepared for further testing with excellent reproducibility and minimal variation in the coated weight per area (SI, Figure S5). For the PU basecoat, the total amount coated (including both abluminal and luminal surfaces) was 29.63 ± 1.77 and 100.14 ± 4.10 µg/mm^2^ for the B-PU_5FU_ and Si-PU_5FU_ stents, respectively. The lower amount coated for the bare stent is attributed to the open cell structure and lack of a supporting substrate, although this did not prevent the formation of uniform, defect-free coatings (Figure 1). For the PEVA topcoat, the total amount coated was 107.23 ± 4.11 and 120.75 ± 7.58 µg/mm^2^ for the B-PU_5FU_-PEVA and Si-PU_5FU_-PEVA stents, respectively. In addition to dip-coating stents, silicon wafers and glass slides were also dip-coated using the exact same solutions and parameters to allow characterization of the coatings.

### 3.2. Characterisation

#### 3.2.1. Photoacoustic FTIR Spectroscopy of Coatings

**Figure 2:**
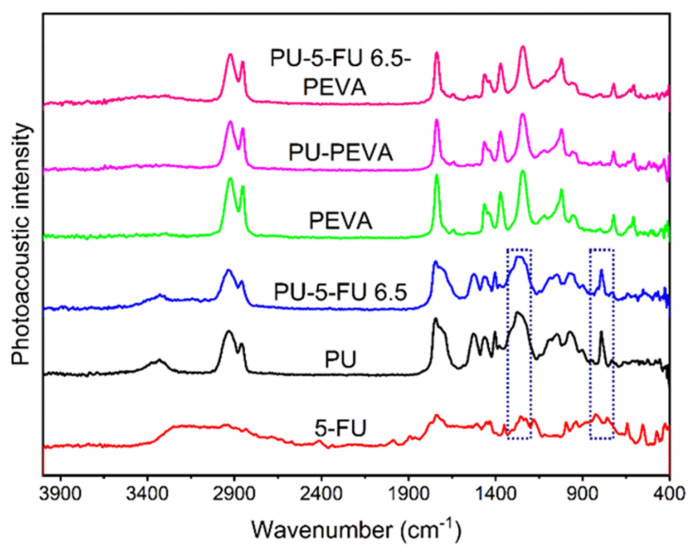
Photoacoustic FT-IR spectra of 5FU and coatings on silicon wafers as indicated. The highlighted regions indicate noticeable peak broadening (left) and peak shoulder (right) resulting from 5FU within the PU coating.

To examine the distribution and interactions of 5FU within the stents, PA-FTIR spectroscopy was performed on coatings prepared on silicon wafers (Figure 2). The spectrum of pure 5FU exhibited characteristic peaks at ~2940, 1740, 1250 and 1230 cm^−1^ consistent with C–H, C=O, C–N and C–F stretching vibrations, respectively, and at ~825 cm^−1^ consistent with C-H deformation [32,59,60,61]. For PU coatings (drug-free), the spectrum revealed peaks at ~3330, 2930, 1740 and 1270 cm^−1^ corresponding to N–H, C–H, C=O and C–O stretching vibrations, and consistent with the reported aliphatic polycarbonate-PU structure [62,63,64,65]. In comparison, the spectrum of the PU_5FU_ coating (basecoat) included all of the characteristic peaks of the PU with a slight broadening of peaks at ~1280 and 814 cm^−1^ due to the presence of 5FU, indicating its successful inclusion into the PU matrix without any apparent changes in their corresponding chemical structures. Subsequently, PEVA coatings were analysed and compared to PU-PEVA (drug-free) and PU_5FU_-PEVA coatings. All coatings produced identical FTIR spectra characteristic of PEVA with ~2920, 1740 and 1240 cm^−1^ stretching vibrations corresponding to C–H, C=O and C–O, respectively [66,67,68]. Importantly, the lack of peaks corresponding to PU or 5FU in the spectra indicated that there is no dissolution of the PU basecoat during the second dip-coating sequence with PEVA and the 5FU does not diffuse into the PEVA topcoat.

#### 3.2.2. X-Ray Diffraction (XRD) of Coatings

**Figure 3:**
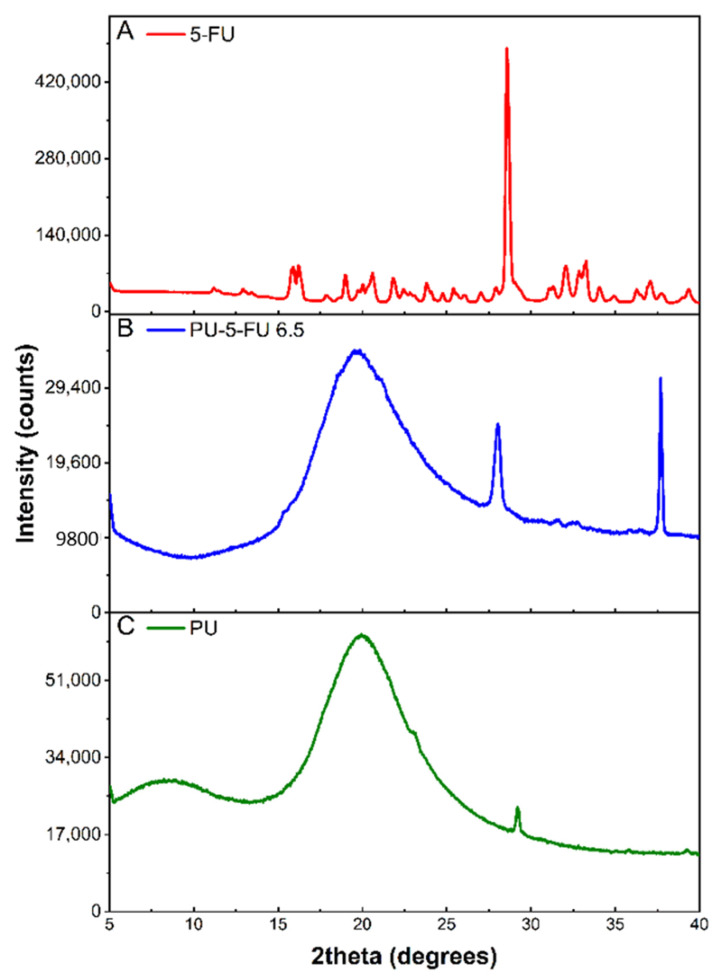
X-ray diffraction patterns of (**A**) pure 5FU, and (**B**) PU_5FU_ and (C) PU coatings.

XRD was used to examine the crystallinity of 5FU within the PU basecoat. XRD patterns of pure 5FU, and PU and PU_5FU_ coatings were recorded (Figure 3) and *d*-spacing values were determined for prominent peaks (SI, Table S4). Pure 5FU revealed a series of characteristic diffraction peaks consistent with those reported previously [42,56,59,61,69,70], with a prominent 2*θ* peak at 28.6° corresponding to a *d*-spacing of 3.123 Å. The diffractogram of the PU coating revealed a broad 2*θ* peak at 19.9° and a low intensity 2*θ* peak at 29.2°, which are consistent with previous literature reports and can be attributed to amorphous and semicrystalline segmented polycarbonate-PUs [43,71,72].

In comparison, the PU_5FU_ coating revealed a broad peak characteristic of the PU matrix and two sharp 2*θ* peaks at 28.1° and 37.7°. The 2*θ* peak at 28.1° (*d*-space = 3.182 Å) in the PU_5FU_ coating was very similar to the dominant 5FU peak (28.6°) and was attributed to the presence of 5FU crystallites within the PU matrix. Broadening of this peak may also be indicative of slight changes in the regular crystalline structure of 5FU or intermolecular interactions with the PU matrix [70]. The decrease in the intensity (28-fold) of the 5FU peaks in the PU matrix compared to the free-drug is unlikely to result from dilution alone, and probably also corresponds to the presence of less and smaller 5FU crystallites [70]. These observations indicate that the majority of 5FU in the PU matrix is in amorphous state along with a small proportion of crystalline drug.

#### 3.2.3. X-Ray Photoelectron Spectroscopy (XPS) of Coatings

**Figure 4:**
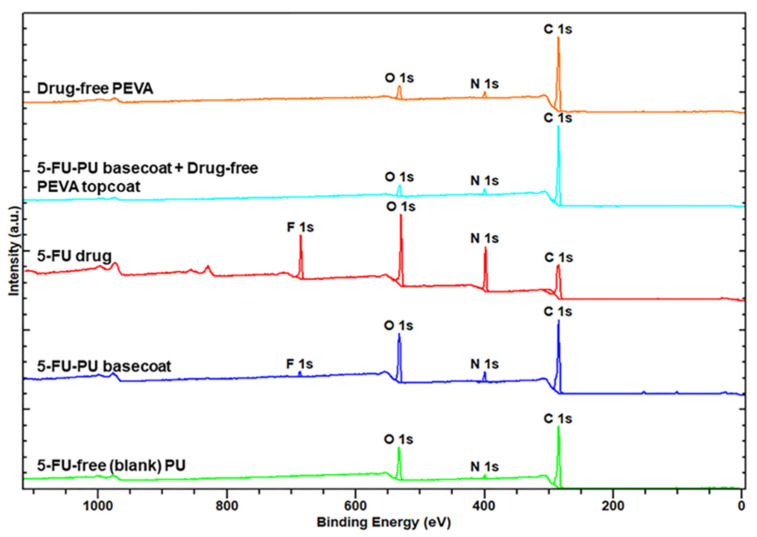
X-ray photoelectron spectra of 5FU and polymer coatings as indicated.

As the 5FU-loaded stents were fabricated via sequential dip-coating steps involving application of the PU_5FU_ basecoat from a THF/DMF binary solvent mixture followed by a PEVA topcoat from a DCM solution it was important to ensure that during the second coating step, the PU basecoat did not swell or loss its integrity leading to 5FU in the PEVA topcoat. Therefore, XPS analysis was performed on polymer coatings and compared to pure 5FU (Figure 4).

As expected, the XPS survey spectrum of pure 5FU revealed peaks corresponding to C, N, O and F atoms [73], whereas the spectra of the PU and PEVA coatings only revealed the presence of C, N and O atoms. In comparison, the spectra of the PU5FU coating displayed a small F 1s peak indicating the successful incorporation of 5FU into the PU matrix and supporting the FTIR results (Figure 2). Importantly, the F 1s peak was absent from the spectra of the PU_5FU_-PEVA coating, indicating that the 5FU does not leach out of the PU layer (to a detectable level) during the second dip-coating step.

#### 3.2.4. Coating Surface Topography and Thickness by SEM

**Figure 5:**
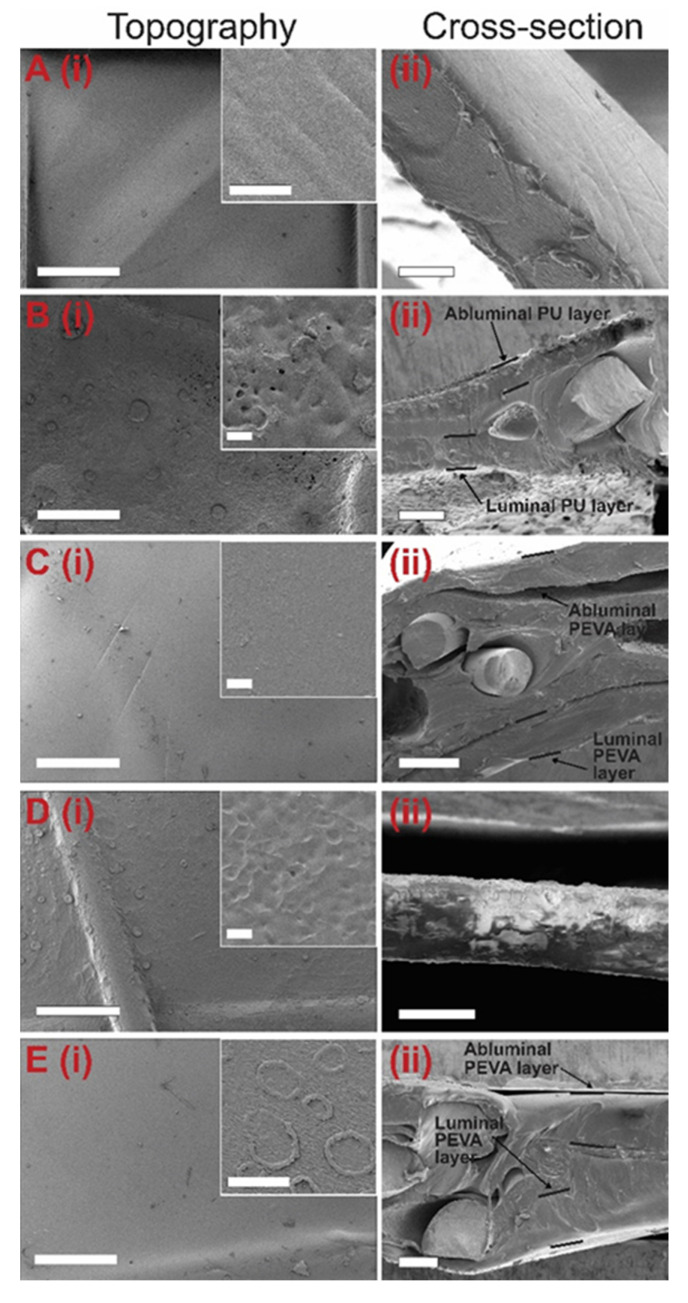
Representative scanning electron microscopy (SEM) images of the (i) abluminal topography (insets show high magnification images) and (ii) cross-section of coated stents before drug release: (**A**) Si-covered nitinol stent with no polymer coating, and (**B**) Si-PU_5FU_, (**C**) Si-PU_5FU_-PEVA, (**D**) B-PU_5FU_, (**E**) B-PU_5FU_-PEVA stents. Scale bars for A-E(i) = 500 microns, A-E(i) inset = 10 microns, A(ii) = 10 microns, B(ii) = 100 microns, C(ii) = 200 microns, D(ii) = 50 microns and E(ii) = 100 microns.

The surface morphology of DES coatings can influence the drug release kinetics and therapeutic response in vitro and in vivo [28,74]. Furthermore, the coating thickness of DESs needs to be precisely controlled to allow optimal delivery and deployment of DESs using delivery catheter systems [24,27]. Therefore, to assess the surface topography (abluminal) and determine the thickness of each coated layer on the stents, SEM imaging was performed on the different layers (Figure 5 and SI, Figures S6–S9).

SEM imaging of the silicone-membrane stents revealed a smooth silicone surface with a thickness of 15.51 ± 0.73 µm (Figure 5A). Following coating with the 5FU-loaded PU, the Si-PU_5FU_ stent surface presented with small pores predominately <5 µm in diameter and irregular aggregates that were attributed to the formation of 5FU crystallites (Figure 5B). This was further confirmed by the absence of the latter in drug-free Si-PU stent surfaces (SI, Figure S6). Cross-sectional imaging of the Si-PU_5FU_ stents provided average thickness values for the PU abluminal and luminal coatings of 48.62 ± 0.62 and 66.20 ± 0.92 µm, respectively. Subsequent coating with PEVA resulted in Si-PU_5FU_-PEVA stents with relatively smooth surfaces, and PEVA abluminal and luminal coating thicknesses of 133.81 ± 5.54 and 134.24 ± 5.61 µm, respectively (Figure 5C).

Coating of the bare nitinol stents with the 5FU-loaded PU resulted in B-PU_5FU_ stent surfaces with similar features to those observed for the Si-PU_5FU_ stents (Figure 5D and SI, Figure S8). The thickness of the PU coating for the B-PU_5FU_ stents was determined to 49.15 ± 1.03 µm. Subsequent coating with PEVA resulted in B-PU_5FU_-PEVA stents with apparently smooth surfaces, although upon higher magnification imaging wrinkles and ring-like structures (~5–10 µm diameter) were observed (Figure 5E). While the latter may result from the formation of bubbles during solvent evaporation, there absence in the Si-PU_5FU_-PEVA stents suggests that the thickness of the underlying basecoat and its susceptibility to deformation may affect the PEVA topcoat. This was further supported by macroscopic images that revealed the formation of wrinkles in the PEVA topcoat for the B-PU_5FU_-PEVA stents, but not the Si-PU_5FU_-PEVA stents (SI, Figure S10). As reported previously, wrinkles commonly emerge in double-coating processes that involve organic solvents in the second coating cycle [74,75]. Thus, the observed differences may result from deformation of the PU coating on B-PU_5FU_-PEVA stents due to the thinner and unsupported PU coating. Nevertheless, the PEVA abluminal (121.31 ± 13.83 µm) and luminal coating (148.94 ± 18.62 µm) thicknesses for the B-PU_5FU_-PEVA stents were similar to those observed for the Si-PU_5FU_-PEVA stents.

#### 3.2.5. Thermal Analysis of Coatings

**Figure 6:**
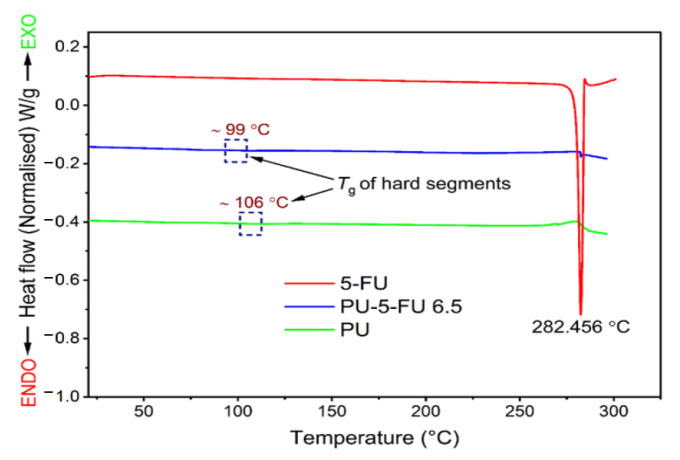
Differential scanning calorimetry thermograms (first heating cycle) recorded at a heating rate of 10 °C/min for pure 5FU, and PU and PU_5FU_ films. The rectangular regions show the hard-segment glass transition temperatures at ~106 and ~99 °C for the PU and PU_5FU_ films, respectively.

DSC was used to investigate the presence of 5FU crystallites within the PU matrix and possible changes in the thermal properties of the PU after drug incorporation. The thermogram of pure 5FU revealed a sharp endothermic peak at 282 °C corresponding to its melting (*Tm*) and degradation temperatures (*Td*) (Figure 6) [32,59,61], whereas the thermogram of PU film displayed a glass transition temperature (*Tg*) of ~106 °C, which is consistent with interactions between hard urethane segments [62,76]. In comparison, the thermogram of PU_5FU_ films revealed a small, sharp endothermic peak at 285 °C consistent with the 5FU *Tm* and a *Tg* at ~99 °C. These results indicate the presence of 5FU crystallites within the PU matrix, corroborating the XRD (Figure 3) and SEM (Figure 5) results, although the actual amount cannot be accurately determined and 5FU may be present in both an amorphous and crystalline forms. The reduction in the *Tg* of the PU matrix upon incorporation of 5FU suggests that some 5FU may be involved in disrupting the polymer-polymer interactions, possibly through the formation of hydrogen-bonding with urethane segments.

**Figure 7:**
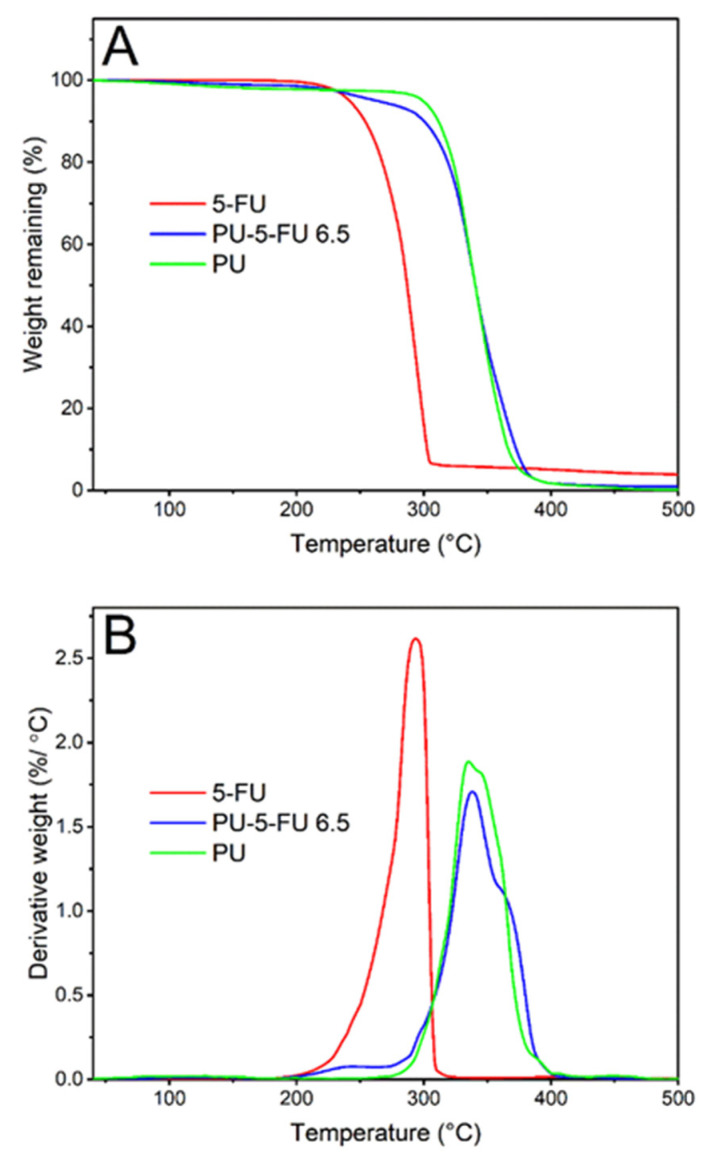
Thermogravimetric (**A**) thermograms recorded between 40 to 500 °C at a heating rate of 10 °C/min and (**B**) derivative weight curves for pure 5FU, and PU and PU_5FU_ films.

TGA was performed to investigate the thermal stability of 5FU within the PU matrix (Figure 7). The thermogram and corresponding derivative weight loss (DTG) curve for pure 5FU implies that the onset of degradation occurs at ~200 °C with a peak maxima at ~293 °C [69,77]. For the PU film, the decomposition onset temperature and peak maxima were observed at ~282 °C and 335 °C, respectively [72,78]. In contrast, the PU_5FU_ film displayed a slight weight decrease (~6.6%) between 200 and 293 °C corresponding to decomposition of 5FU, followed by decomposition of the PU matrix. Despite slight overlap in the degradation events, the degradation of 5FU within the PU matrix based on weight loss correlates well with the original loading of the PU coating. Nevertheless, these results indicate that there was no notable change in the thermal stability of the 5FU due to inclusion in the PU polymer matrix.

#### 3.2.6. In Vitro Drug Release

**Figure 8:**
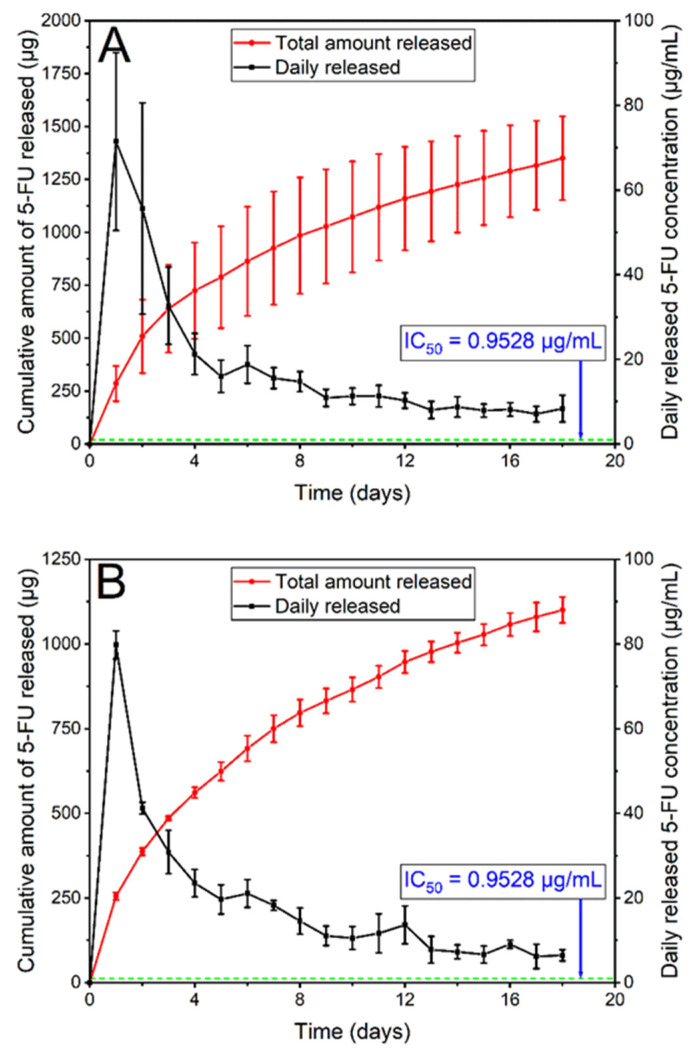
In vitro release of 5FU from (**A**) Si-PU_5FU_-PEVA and (**B**) B-PU_5FU_-PEVA stent sections over 18 d in 4 mL and 3.2 mL of 10% *v/v* FBS-supplemented sterile RPMI-1640 medium, respectively. Data expressed as mean (*n* = 3) ± SD.

In vitro release of 5FU from the stents was conducted by submerging cut stent sections (6 × 6 and 10 × 10 mm each Si-PU_5FU_-PEVA and B-PU_5FU_-PEVA stent section, respectively) in 10% *v/v* FBS-supplemented sterile RPMI-1640 medium (FBS-RPMI), and the release media was collected at each timepoint to determine the concentration of released 5FU (Figure 8) and for subsequent cell experiments. The solubility of 5FU in the FBS-RPMI medium was initially determined (11.26 ± 1.30 mg/mL; *n* = 4) to ensure the maintenance of sink conditions. For both stent types asymptotic release profiles were observed, with an initial burst release over the first few days followed by a consistent decrease of drug release over consecutive days. The higher cumulative release observed from the Si-PU_5FU_-PEVA stents over 18 d (1351 µg as compared to 1101 µg for the B-PU_5FU_-PEVA stents) was attributed to the higher amount of 5FU present in those stents.

### 3.3. In Vitro Anticancer Activity of the 5FU-Loaded GI Stents

Release studies in FBS-RPMI media revealed that the Si-PU_5FU_-PEVA and B-PU_5FU_-PEVA stents exhibited sustained 5FU release for at least 18 consecutive days (Figure 8). In order to mimic the possible clinical scenario of time-course drug release from the stents, a non-cumulative approach was considered suitable for cytotoxicity determinations [50]. Therefore, all 5FU-released samples were collected separately on a daily basis and selected timepoints (day 1, 7, or 14) were used for cellular evaluations without mixing with any other sample replicates, either from the same day or from the other days.

To evaluate the anticancer activity of the 5FU released from the Si-PU_5FU_-PEVA and B-PU_5FU_-PEVA stents, human colon carcinoma (HCT-116) cells were used. Initially, the cytotoxicity of pure 5FU was determined via MTT assay by incubating the cells with various drug concentrations ranging from 0.1 to 195.1 µg/mL for 72 h, which showed a dose-dependent reduction in viability and provided an IC_50_ value of 0.95 µg/mL (SI, Figure S11). Importantly, the IC50 value is significantly smaller than the 5FU concentration released from the stents for each day of the release study (Figure 8). Therefore, it is conceivable that the Si-PU_5FU_-PEVA and B-PU_5FU_-PEVA stents would provide controlled in vivo intracolonic release of therapeutic concentrations of 5FU over a prolonged time period.

#### 3.3.1. Cytotoxicity of Stent Released 5FU

**Figure 9:**
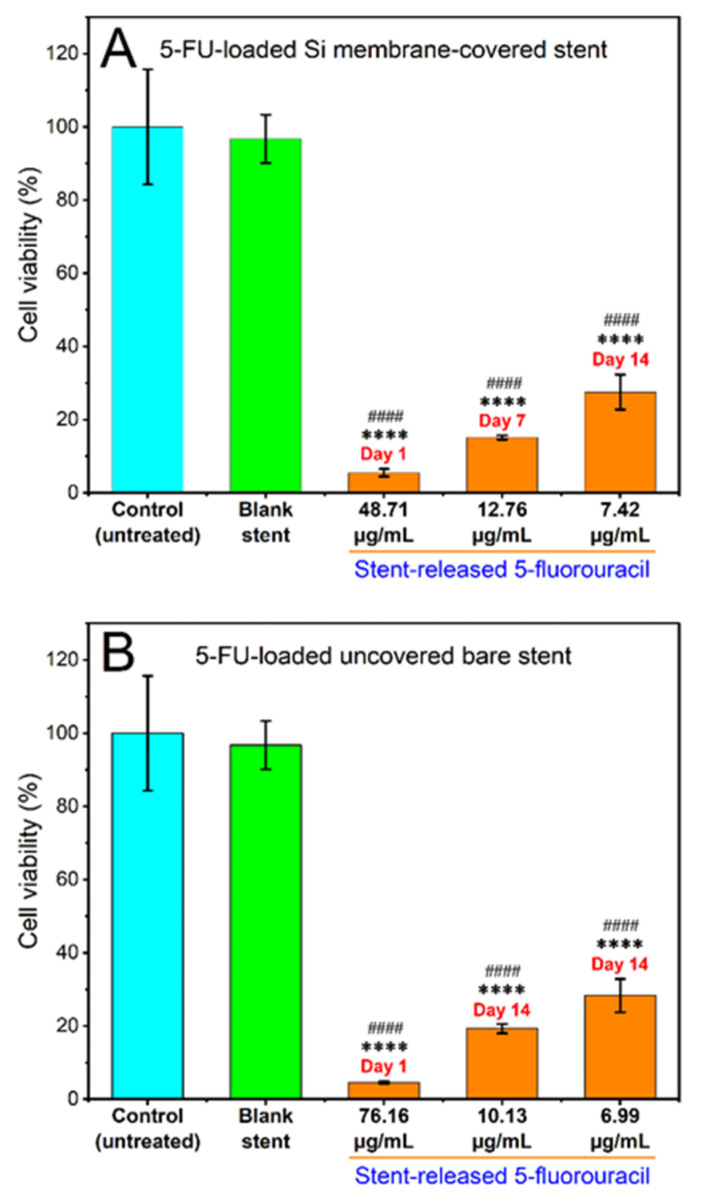
Effects of various concentrations of 5FU released over day 1, 7 or 14 from the (**A**) Si-PU_5FU_-PEVA and (**B**) B-PU_5FU_-PEVA stent sections on the viability of HCT-116 human colon cancer cells treated for 72 h, determined by MTT assay. Results from at least three replicate measurements are given as mean ± SD. *p*-values were calculated using one-way analysis of variance (ANOVA). **** *p* < 0.0001 and #### *p* < 0.0001 compared to the untreated control and blank (drug-free) stent sections, respectively.

The cytotoxicity of the released 5FU from the Si-PU_5FU_-PEVA and B-PU_5FU_-PEVA stents (*n* = 3 for each) was subsequently assessed against HCT-116 cells after incubation for 72 h using the MTT assay (Figure 9). To compare the cytotoxic effect between the stent-released 5FU and pure 5FU (positive control), cell viability (%) data for 5FU drug concentrations that were the same to each of the stent-released 5FU concentrations were calculated from the IC_50_ curve (SI, Figures S11 and S12) [79]. In addition, media collected from drug-free Si-PU-PEVA and B-PU-PEVA stents sections were also included in the assay as blank controls.

The cell viability for drug-free blank sections was ~97% compared to the non-treated (media) controls, which excludes the possibility of cytotoxicity from the PU-PEVA stent platform. For both 5FU-loaded stent types there was a clear trend of concentration-dependent reduction of cell viability following 72 h of exposure, with statistically significant differences observed between the stent-released 5FU concentrations and the non-treated (media) or drug-free (blank) controls (Figure 9). In comparison to pure 5FU drug concentrations, all stent-released 5FU concentrations showed similar inhibition of cell proliferation (SI, Figure S12). Since the stent-released 5FU concentrations were not exactly the same as the pure 5FU drug concentrations (positive control) used in constructing the 5FU dose-response curve, no statistical significance was determined between the cell viability (%) data of the stent-released 5FU and pure 5FU drug. However, these results demonstrate that the proliferation of HCT-116 cancer cells was effectively inhibited by the stent-released 5FU; hence, the fabricated 5FU-loaded stents are capable of maintaining cytotoxic activities in vitro throughout the 14-day release period without significant loss of biological activity.

#### 3.3.2. Cell Cycle Analysis and Detection of Apoptosis by Flow Cytometry

**Figure 10:**
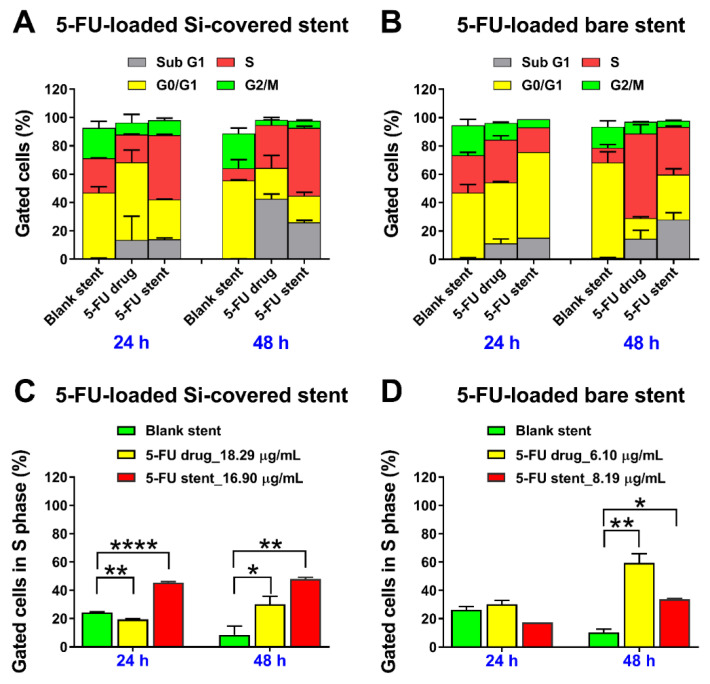
In vitro effects of 5FU released over two days from the (**A**,**C**) Si-PU_5FU_-PEVA and (**B**,**D**) B-PU_5FU_-PEVA stent sections, on (**A**,**B**) different phases or in the (**C**,**D**) S phase of HCT-116 human colon cancer cells treated for 24 and 48 h; evaluated using flow cytometry. Cell cycle data are presented without subtracting the value of untreated control (media), as mean ± SD from two independent experiments. **** *p* < 0.0001, ** *p* < 0.01 and * *p* < 0.05 compared to blank (drug-free) bilayer coated nitinol control stent.

Subsequently we investigated whether the concentrations of 5FU released from the Si-PU_5FU_-PEVA and B-PU_5FU_-PEVA stents affected the cell cycle distribution of HCT-116 cells after 24 or 48 h incubation, compared with that of the pure drug (Figure 10 and SI, Figure S13). Cell cycle analysis is a valuable tool that provides information on different phases of the eukaryotic cell cycle (G0/G1, S, and G2/M) depending upon the DNA content [48]. It is well-known that the initiation of cell division is regulated at the G1 phase [48] and the replication of DNA is progressed in the S phase [12]. Cell cycle checkpoints are rigorous quality control steps or mechanisms that function in maintaining the integrity of DNA and thus ensure the fidelity of cell division [12,80]. There are a number of different cell cycle checkpoints (e.g., G1 at G1/S transition, G2 at G2/M transition, etc.) and these checkpoints examine whether the relevant processes at each phase of the ongoing cycle have been completed accurately before progressing to the next phase. In case of any detection of DNA damage or replication errors by the checkpoints would halt or delay the cell cycle progression and permit the cell to repair the DNA damage itself before entering into the next phase-the failure of which could result in the induction of apoptosis, senescence, or mitotic catastrophe [12,80]. However, the fluoropyrimidine analogue 5FU [29] is an antimetabolite chemotherapeutic which exerts cytotoxicity primarily through impairment of DNA replication during the S phase of the cell cycle [12,81] and its cytotoxic activity is both dose and time-dependent [82].

For the drug-free (blank) Si-PU-PEVA and B-PU-PEVA stents no noticeable accumulation of cell populations in any specific phase of the cycle was observed after exposure for up to 48 h (Figure 10A,B, respectively). In comparison, treatment with 5FU released from the Si-PU_5FU_-PEVA and B-PU_5FU_-PEVA stent sections (equivalent to 16.9 and 8.19 µg/mL, respectively) for up to 48 h resulted in an increase in the percentage of cells undergoing S cycle arrest (Figure 10C,D, respectively). For the Si-PU_5FU_-PEVA-released 5FU (16.90 µg/mL), the S population increased from 45.4% at 24 h to 48.0% at 48 h, indicating that a longer incubation time did not effectively increase the S cycle arrest. In contrast, the B-PU_5FU_-PEVA-released 5FU (8.19 µg/mL) had a more delayed effect in S cycle arrest with an approximate doubling of the S phase population from 17.4% at 24 h to 33.7% at 48 h. These results suggest that the higher concentration of 5FU (16.90 µg/mL) released from the Si-PU_5FU_-PEVA stents had acute cytotoxicity compared to the more prolonged effect of a lower 5FU concentration (8.19 µg/mL) released from the B-PU_5FU_-PEVA stents. Interestingly, the effect of stent-released 5FU in the S cycle arrest was found to be somewhat proportional to its concentration after 24 h of exposure, irrespective of the stent type. However, similar cell cycle profiles were observed in HCT-116 cells at 24 or 48 h following exposure to pure 5FU drug at similar concentrations (18.29 and 6.10 µg/mL). These results confirmed that the 5FU released from the 5FU-loaded stents had both concentration- and time-dependent effects as consistent with its intrinsic nature [12,82].

**Figure 11:**
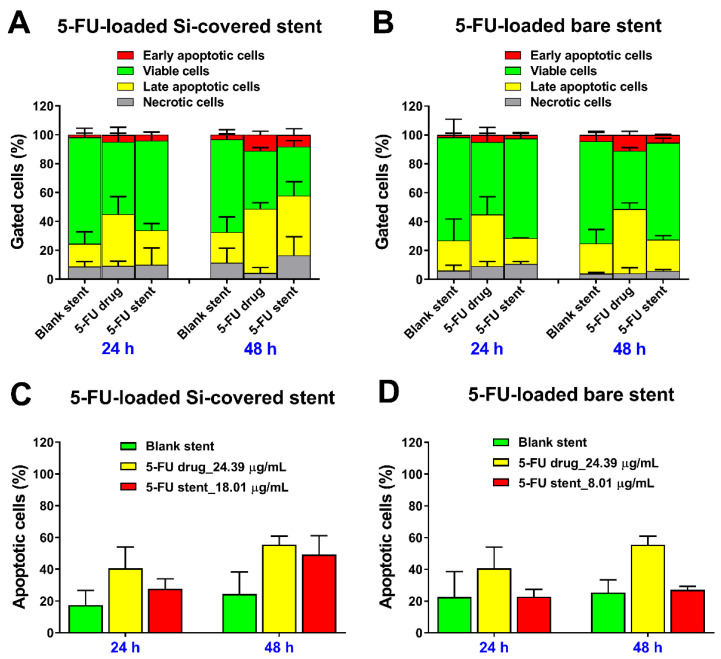
Evaluation of in vitro anticancer effect of 5FU released from the (**A**,**C**) Si-PU_5FU_-PEVA and (**B**,**D**) B-PU_5FU_-PEVA stent sections at 24 and 48 h on HCT-116 human colon cancer cells; determined by flow cytometric analysis. (**C**,**D**) Total apoptotic cells were calculated by adding the early and late apoptotic cells. All data represent mean ± SD from at least two replicate measurements, without subtracting the value of untreated control (media).

Furthermore, a substantial accumulation of cells in the Sub G1 phase was detected at 48 h for both the stent-released 5FU samples (14.1% at 24 h against 26.0% at 48 h for Si-PU_5FU_-PEVA stent sample; and 15.2% at 24 h against 28.1% at 48 h for B-PU_5FU_-PEVA stent sample), which provided evidence of cell death (Figure 10). Predominantly, cell death in multicellular organisms occurs via apoptosis or necrosis [83], with 5FU being reported to induce apoptosis [84]. Therefore, the mechanism of cell death induced by the 5FU released from the stents was further investigated using the flow cytometry-based annexin V-FITC/PI dual staining assay following treatment of HCT-116 cells for a period of 24 and 48 h (Figure 11 and SI, Figure S14). Additionally, drug-free (blank) stent samples, and pure 5FU (24.39 µg/mL) were included in the apoptosis experiments for comparison purposes.

As expected, for both of the drug free stent controls, the majority of cells were viable (~70%) at 24 and 48 h, with ~20% in the late apoptotic stage. In comparison, treatment with pure 5FU (24.39 µg/mL) or 5FU released from the Si-PU_5FU_-PEVA and B-PU_5FU_-PEVA stents sections (18.01 and 8.01 µg/mL, respectively) for up to 48 h incubation resulted in an increase in both early and late apoptotic cells in a concentration- and time-dependent fashion (Figure 11A,B). However, the 5FU released from the Si-PU_5FU_-PEVA stent sections resulted in a higher number of apoptotic cells (early and late stage) at 48 h as compared to 5FU released from the B-PU_5FU_-PEVA stent sections (Figure 11C,D). Taken together, these results demonstrated that HCT-116 cells are dependent on 5FU activity for survival and both the 5FU-loaded stents had a clear 5FU induced anticancer effect through the apoptosis mechanism as consistent with previous literature [12,81,84].

## 4. Conclusions

Two drug-eluting stent systems were successfully fabricated using sequential dip-coating of either bare or silicone-membrane self-expanding metal colonic stents with a 5-fluorouracil (5FU)-loaded (6.5% *w/w*) polyurethane (PU) basecoat and poly(ethylene-*co*-vinyl acetate) (PEVA) topcoat. Optimisation of the coating formulations allowed the hydrophilic 5FU to be successfully incorporated into a hydrophobic PU matrix, and further coating with PEVA allowed the drug-release to be controlled. Physiochemical characterization of the stents and coatings revealed that the 5FU is incorporated into the polyurethane matrix uniformly, and is likely to be present in both amorphous and crystalline forms. Imaging of the stents further confirmed these findings and allowed the thickness of the coatings to be determined, which were similar for both types of stents. In vitro drug release studies revealed the sustained release of 5FU over a period of 18 days, and cell viability, cell cycle and cell apoptosis assays confirmed that the released 5FU from the stents had similar anticancer activity in vitro compared to pure 5FU drug against human colon cancer cells. Taken together, the results of the physicochemical and biological characterisation studies are promising and highlight the potential of these stents for the treatment of colorectal cancer and its related stenosis/obstructions.

## Data Availability

The data presented in this study are available at University of South Australia storage system.

## Supplementary Materials

The following are available online at https://www.mdpi.com/1999-4923/13/1/17/s1, Figure S1. ^1^H-NMR spectrum of ChronoFlex AL (polyurethane, PU); Figure S2. ^13^C-NMR spectrum of ChronoFlex AL (polyurethane, PU); Figure S3. (A) Chemical structure of 5-fluorouracil (5FU), (B) generic structure of ChronoFlex AL (polyurethane, PU), and (C) chemical structure of poly(ethylene-*co*-vinyl acetate) (PEVA); Figure S4. Desktop dip-coater used to prepare coated stent; Figure S5. Comparison of percentage weight increase and amount of coating per unit area between the corresponding basecoat and topcoat layers of the 5FU-loaded stents; Figure S6. Top-view field emission scanning electron microscopy images of representative abluminal surface morphologies of the Si-covered stents with various coatings before drug release; Figure S7. Representative cross-section scanning electron microscopy images showing coating thickness on the surface of the Si-covered stents with various coatings before drug release; Figure S8. Top-view field emission scanning electron microscopy images of representative abluminal surface morphologies of the bare stents with various coatings before drug release; Figure S9. Representative cross-section scanning electron microscopy images showing coating thickness on the surface of the coated bare stent before drug release; Figure S10. Representative luminal top-view optical images showing the PEVA topcoat surfaces of (A) Si-PU_5FU_-PEVA and (B) B-PU_5FU_-PEVA stents; Figure S11. A representative concentration-response curve showing the inhibition of 50% cell viability (IC_50_) for HCT-116 human colon cancer cell line treated with pure 5FU for 72 h by MTT assay; Figure S12. Comparison of cytotoxic effects of different concentrations of 5FU released after day 1, 7 or 14 from (A) Si-PU_5FU_-PEVA and (B) B-PU_5FU_-PEVA stent sections, with the respective 5FU drug concentrations (positive control) on HCT-116 human colon cancer cells treated for 72 h by MTT assay; Figure S13. Cell cycle distribution (%) of HCT-116 human colon cancer cells in different treatment groups at 24 and 48 h; Figure S14. Apoptosis progression in HCT-116 human colon cancer cells after treatment with drug-free Si-PU-PEVA and B-PU-PEVA stents, 5FU drug (positive control) and drug-loaded Si-PU_5FU_-PEVA and B-PU_5FU_-PEVA stents for 24 and 48 h, as analysed by a flow cytometry-based annexin V-FITC/propidium iodide (PI) assay; Table S1. Composition of the stent coating solutions; Table S2. Optimised parameters used for dip-coating the commercial nitinol stents; Table S3. Description/Type of samples used in different characterisation studies of the films and coated stents; Table S4. XRD data of pure 5FU drug and 6.5% *w/w* 5FU-loaded PU monolayer film.
