# Supplementary material for: Development and In Vitro Evaluation of 5-Fluorouracil-Eluting Stents for the Treatment of Colorectal Cancer and Cancer-Related Obstruction
Source: Pharmaceutics. 2020 Dec 24;13(1):17. doi: 10.3390/pharmaceutics13010017 (PMC7823773; doi:10.3390/pharmaceutics13010017)
Supplement: Supplementary file 1 [file pharmaceutics-13-00017-s001.pdf]

pharmaceutics

Supplementary Materials: Development and In Vitro Evaluation of 5-Fluorouracil-Eluting Stents for the Treatment of Colorectal Cancer and Cancer-related Obstruction

Mohammad Arafat, Paris Fouladian, Anthony Wignall, Yunmei Song, Ankit Parikh, Hugo Albrecht, Clive A. Prestidge, Sanjay Garg and Anton Blencowe

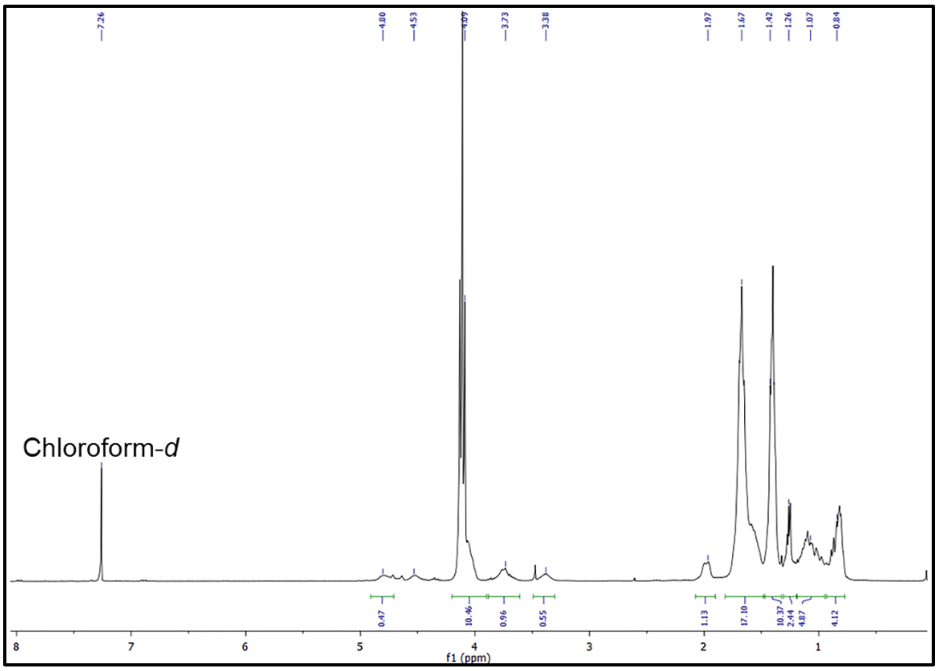

Figure S1. ^{1}H-NMR spectrum of ChronoFlex AL (polyurethane, PU).

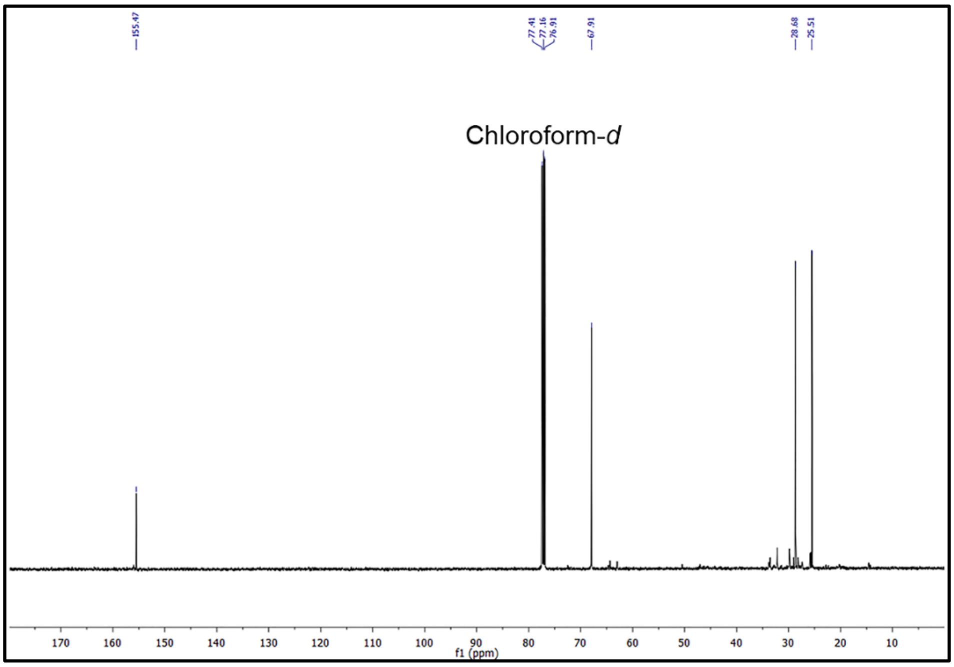

Figure S2. ^{13}C-NMR spectrum of ChronoFlex AL (polyurethane, PU).

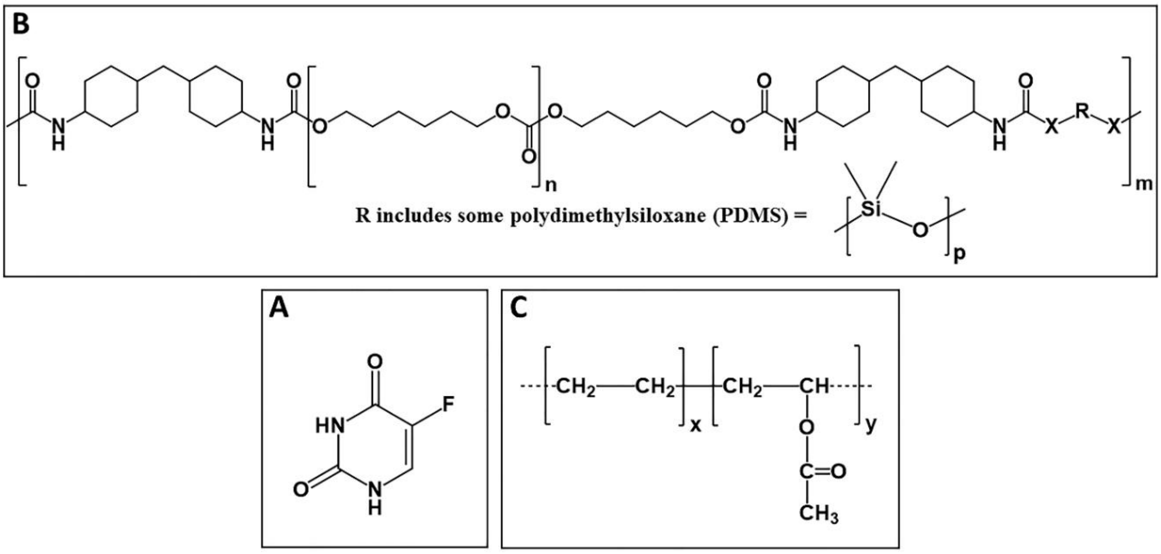

Figure S3. (A) Chemical structure of 5-fluorouracil (5FU), (B) generic structure of ChronoFlex AL (polyurethane, PU), and (C) chemical structure of poly(ethylene-co-vinyl acetate) (PEVA).

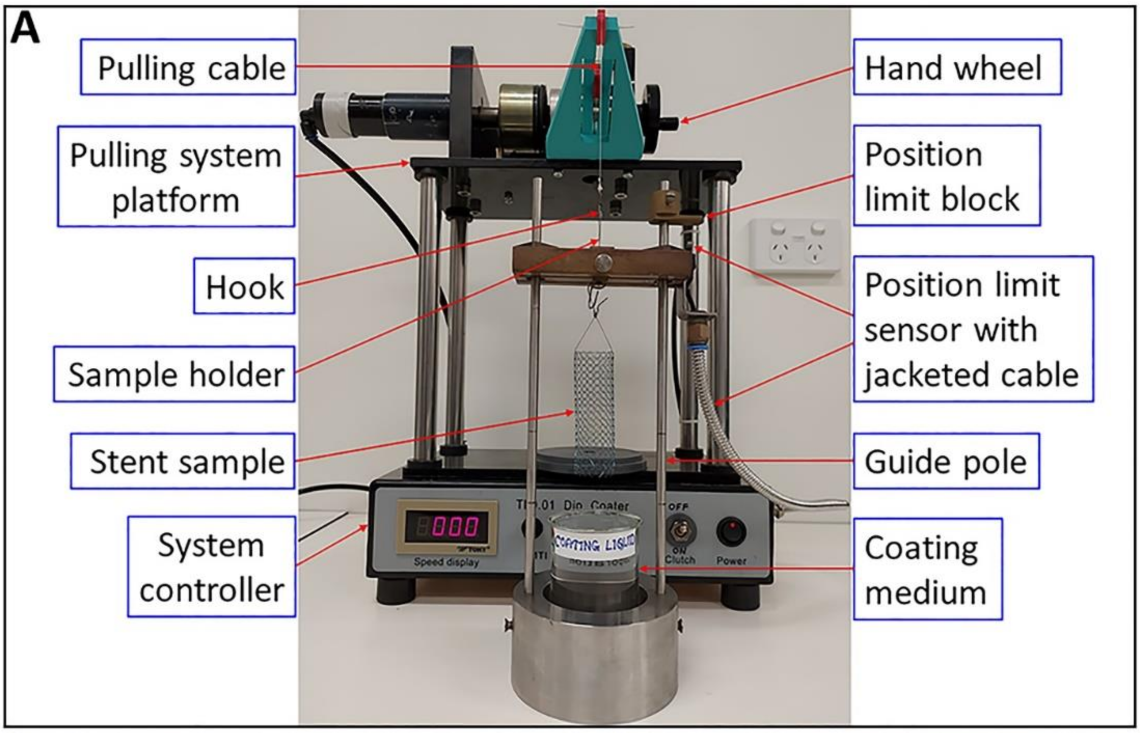

Figure S4. Desktop dip-coater used to prepare coated stent.

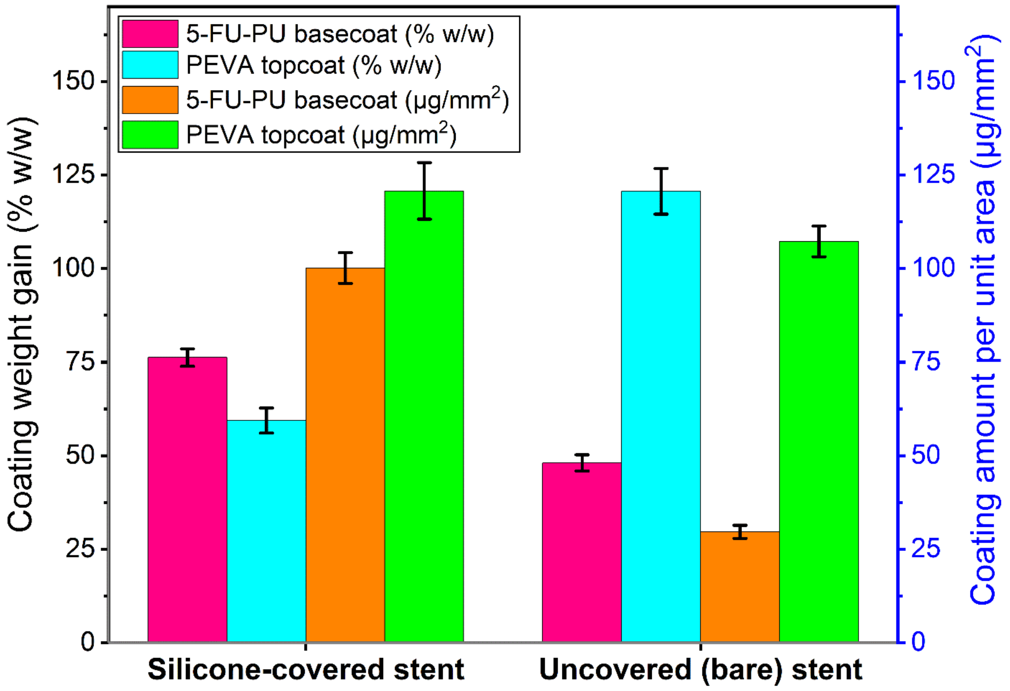

Figure S5. Comparison of percentage weight increase and amount of coating per unit area between the corresponding basecoat and topcoat layers of the 5FU-loaded stents. Area was calculated from both the abluminal and luminal sides of the stents. Data represent mean (n = 10) ± Standard deviation (SD).

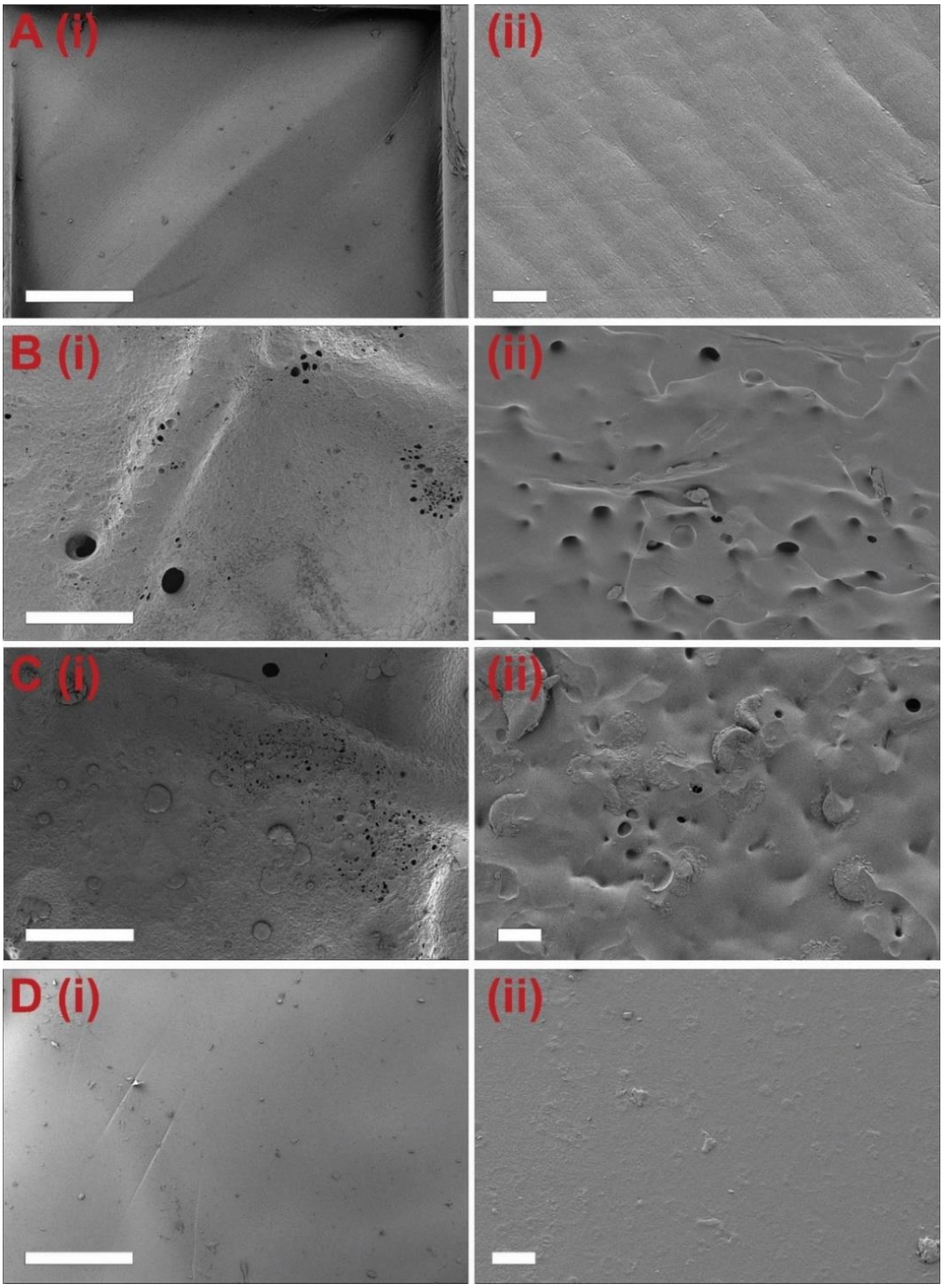

Figure S6. Top-view field emission scanning electron microscopy images of representative abluminal surface morphologies of the Si-covered stents with various coatings before drug release, at (A-D(i)) low and (A-D(ii)) high magnifications: (A) Si-covered stent silicone membrane surface, and (B) **Si-PU**, (C) **Si-PU$_{5FU}$**, (D) **Si-PU$_{5FU}$-PEVA** stent surfaces. Scale bars for A-D(i) = 500 μm, A(ii) = 5 μm, and B-D(ii) = 10 μm.

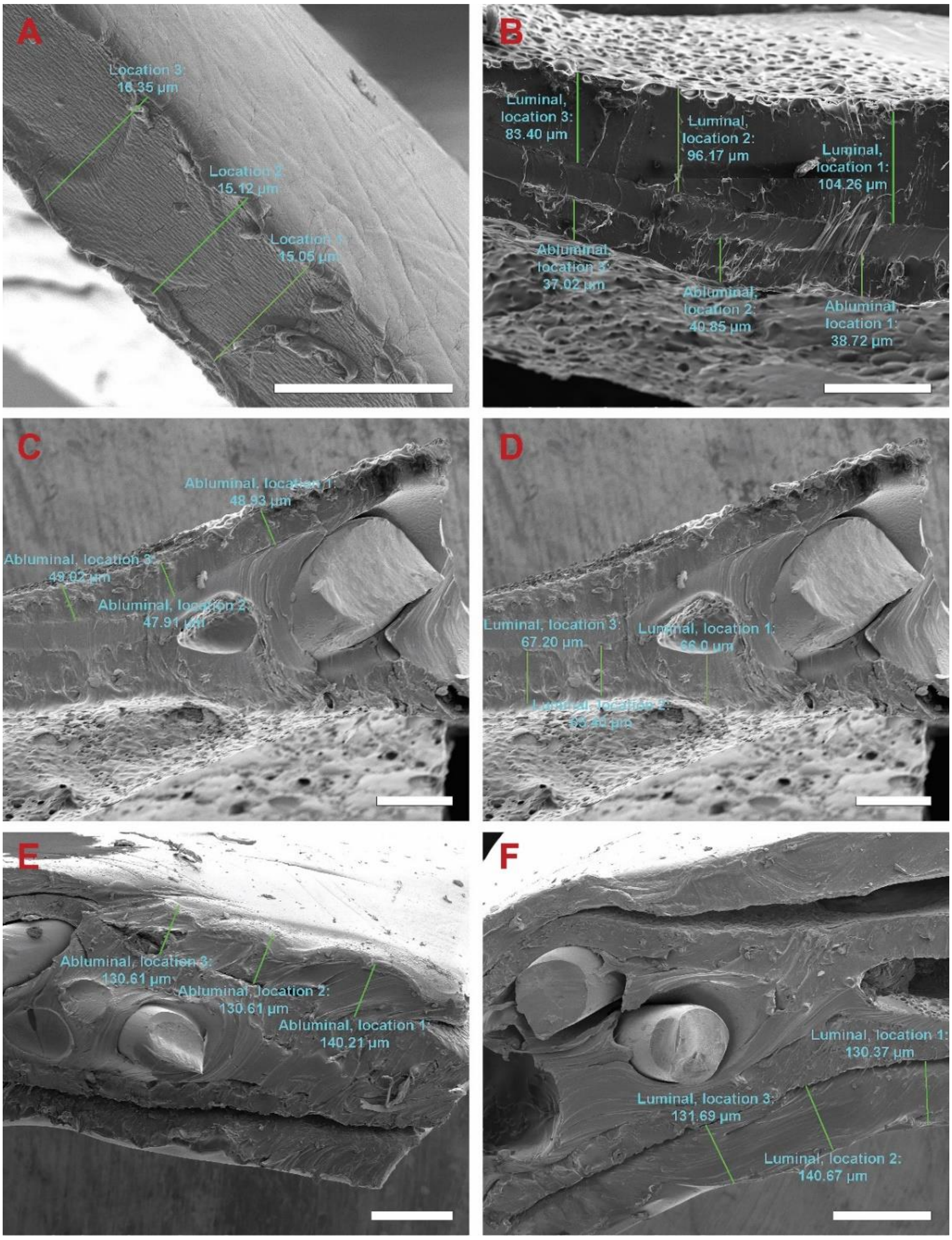

Figure S7. Representative cross-section scanning electron microscopy images showing coating thickness on the surface of the Si-covered stents with various coatings before drug release: (A) Si-covered stent silicone membrane cross-section, and (B) **Si-PU**, (C, D) abluminal and luminal **Si-PU$_{5FU}$**, (E, F) abluminal and luminal **Si-PU$_{5FU}$-PEVA** cross sections with measurements. Scale bars for A = 20 μm, B-D = 100 μm, and E-F = 200 μm.

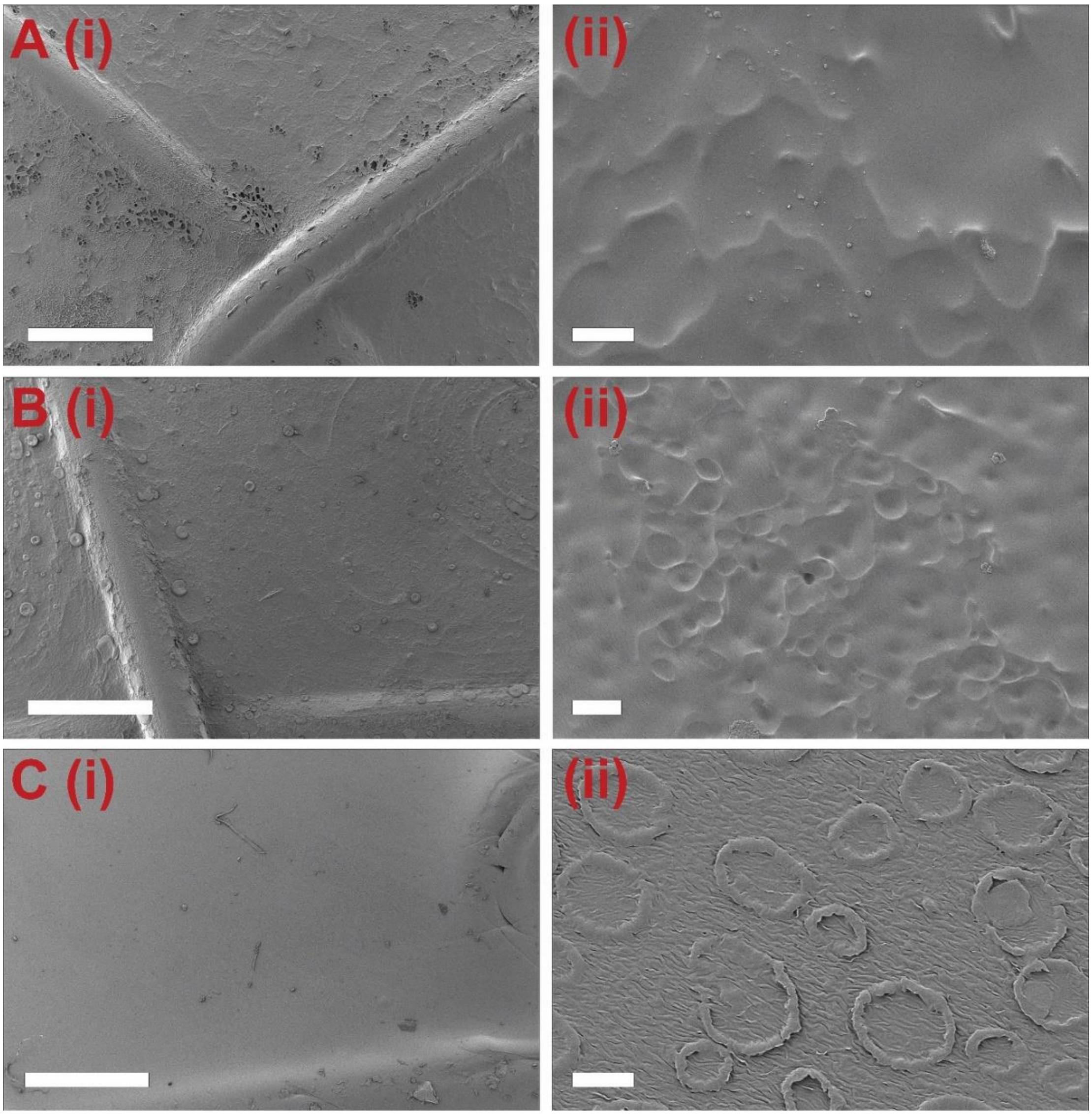

Figure S8. Top-view field emission scanning electron microscopy images of representative abluminal surface morphologies of the bare stents with various coatings before drug release, at (A-C(i)) low and (A-C(ii)) high magnifications: (A) **B-PU**, (B) **B-PU$_{5FU}$**, and (C) **B-PU$_{5FU}$-PEVA** stent surface. Scale bars for A-C(i) = 500 μm, A(ii) = 5 μm, B(ii) = 10 μm, and C(ii) = 5 μm.

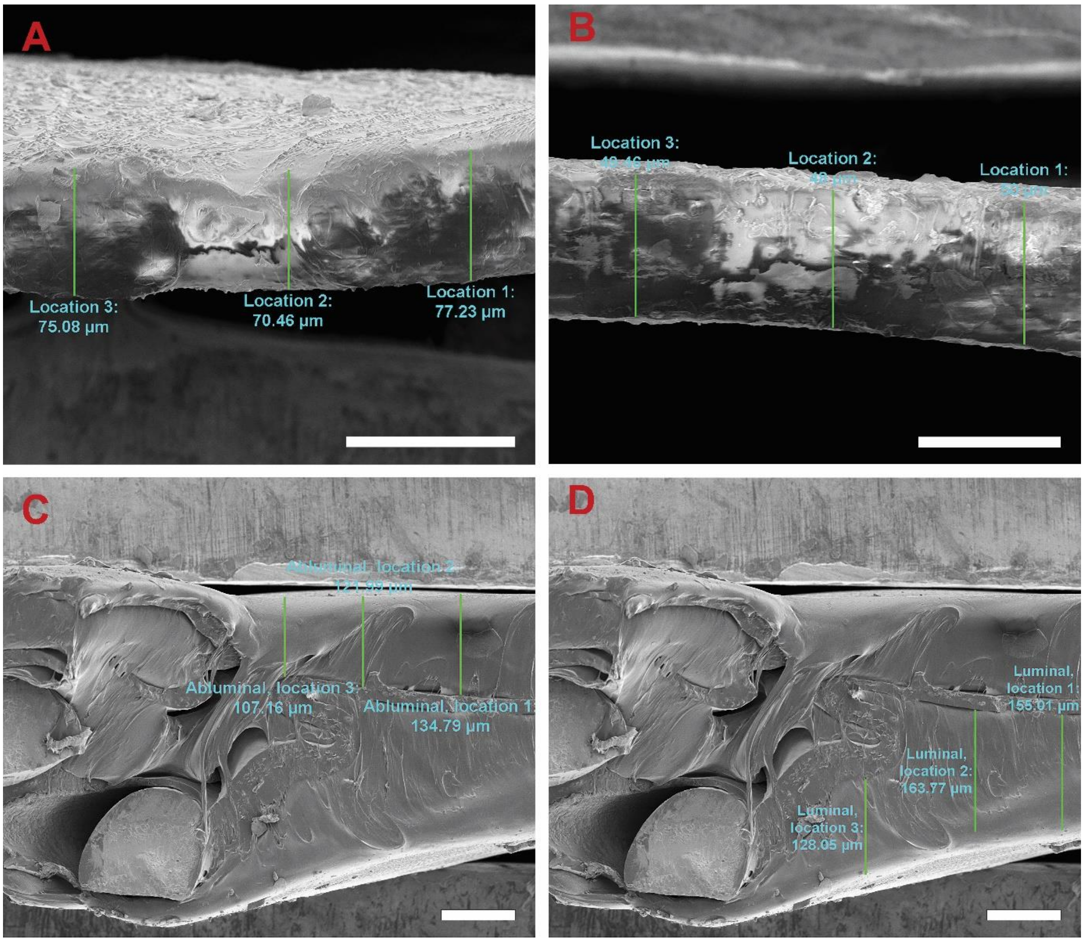

Figure S9. Representative cross-section scanning electron microscopy images showing coating thickness on the surface of the coated bare stent before drug release: (A) **B-PU**, (B) **B-PU$_{5FU}$** (C, D) abluminal and luminal **B-PU$_{5FU}$-PEVA** cross sections with measurements. Scale bars for A-B = 100 µm, C = 50 µm, and D = 100 µm.

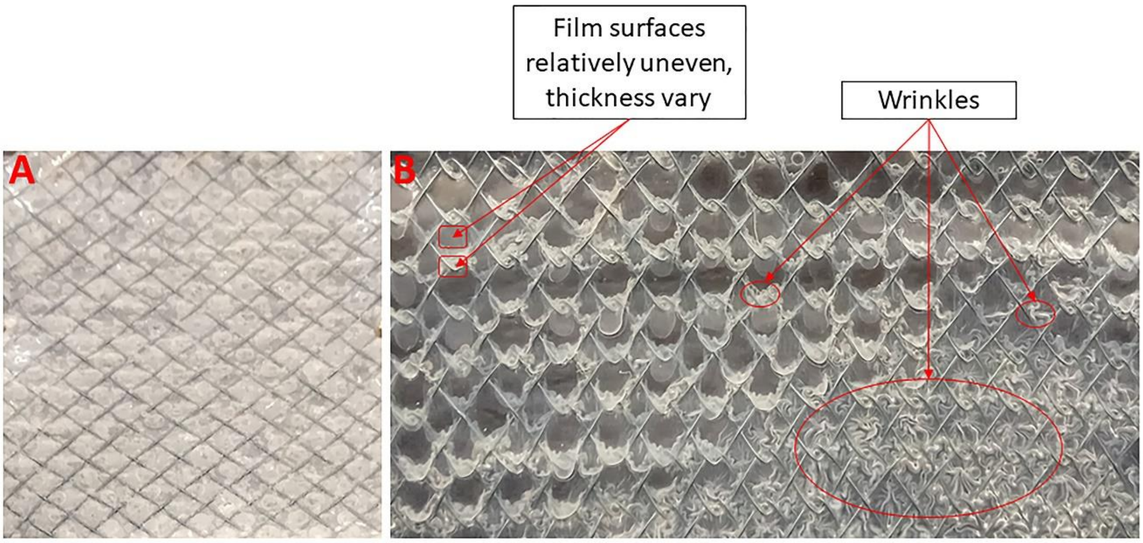

Figure S10. Representative luminal top-view optical images showing the PEVA topcoat surfaces of (A) **Si-PU$_{5FU}$-PEVA** and (B) **B-PU$_{5FU}$-PEVA** stents.

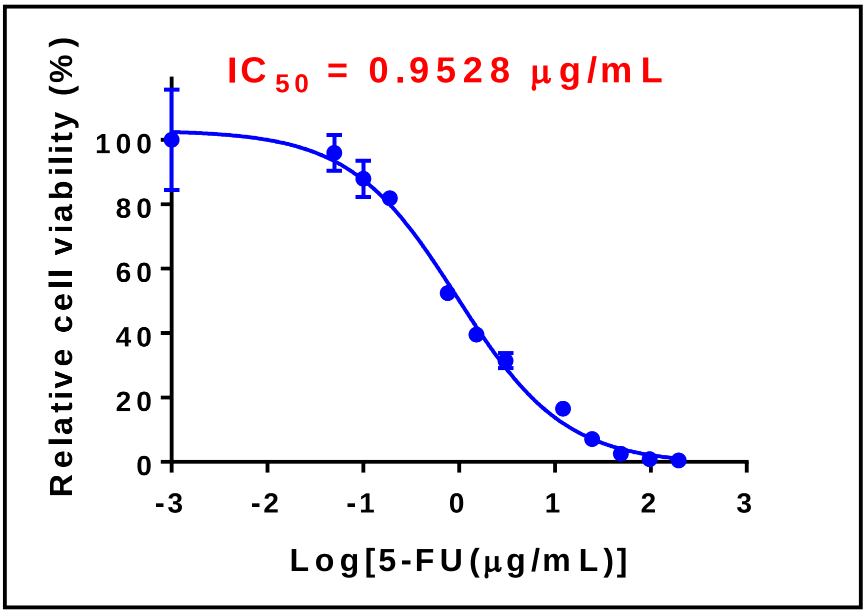

Figure S11. A representative concentration-response curve showing the inhibition of 50% cell viability (IC_{50}) for HCT-116 human colon cancer cell line treated with pure 5FU for 72 h by MTT assay. All experiments were performed in triplicates and results are presented as mean ± SD.

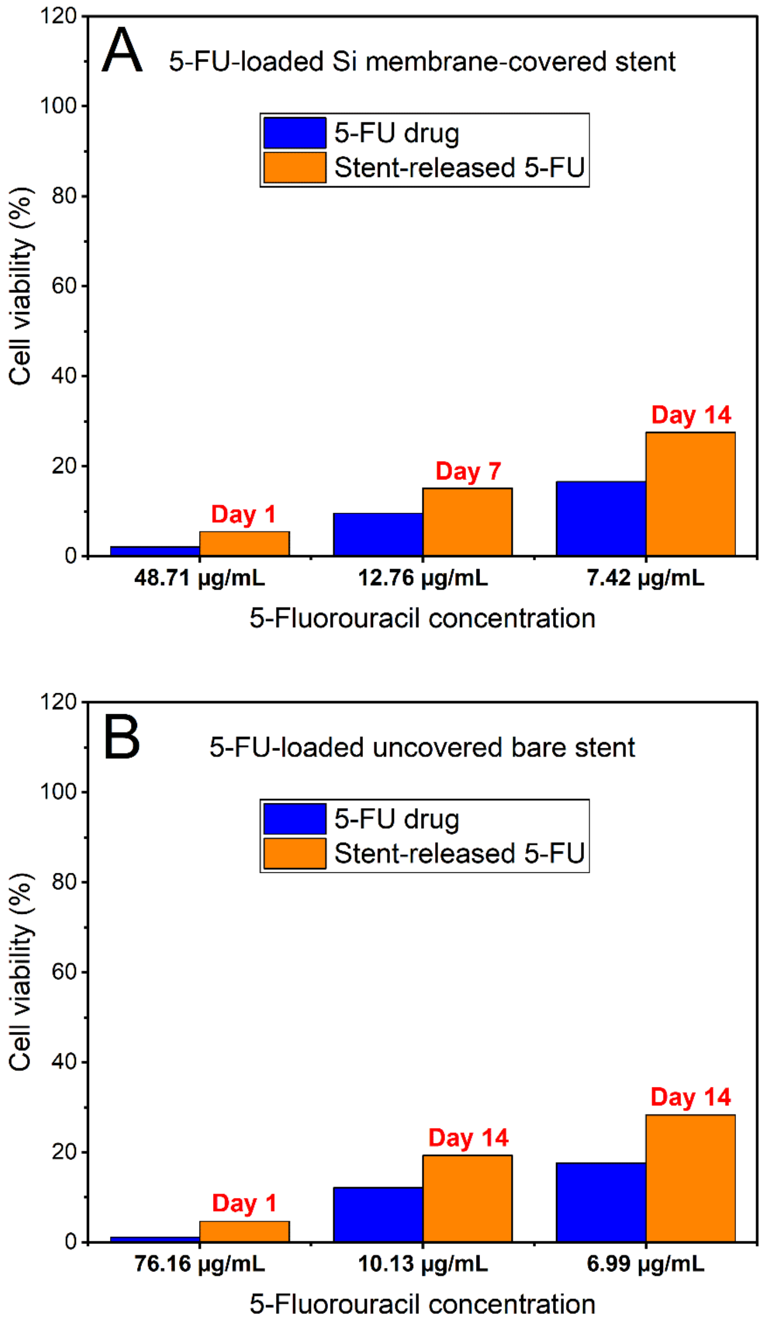

Figure S12. Comparison of cytotoxic effects of different concentrations of 5FU released after day 1, 7 or 14 from (A) **Si-PU$_{5FU}$-PEVA** and (B) **B-PU$_{5FU}$-PEVA** stent sections, with the respective 5FU drug concentrations (positive control) on HCT-116 human colon cancer cells treated for 72 h by MTT assay. The cell viability (%) of the positive controls were calculated from the IC_{50} curve using the equation **Y = Bottom + (Top – Bottom) / (1 + 10^(logIC_{50}) – X) × Hillslope)** from a four-parameter logistic (4PL) curve-fit, while the 5FU coated stent samples are presented as mean of at least three replicate measurements.

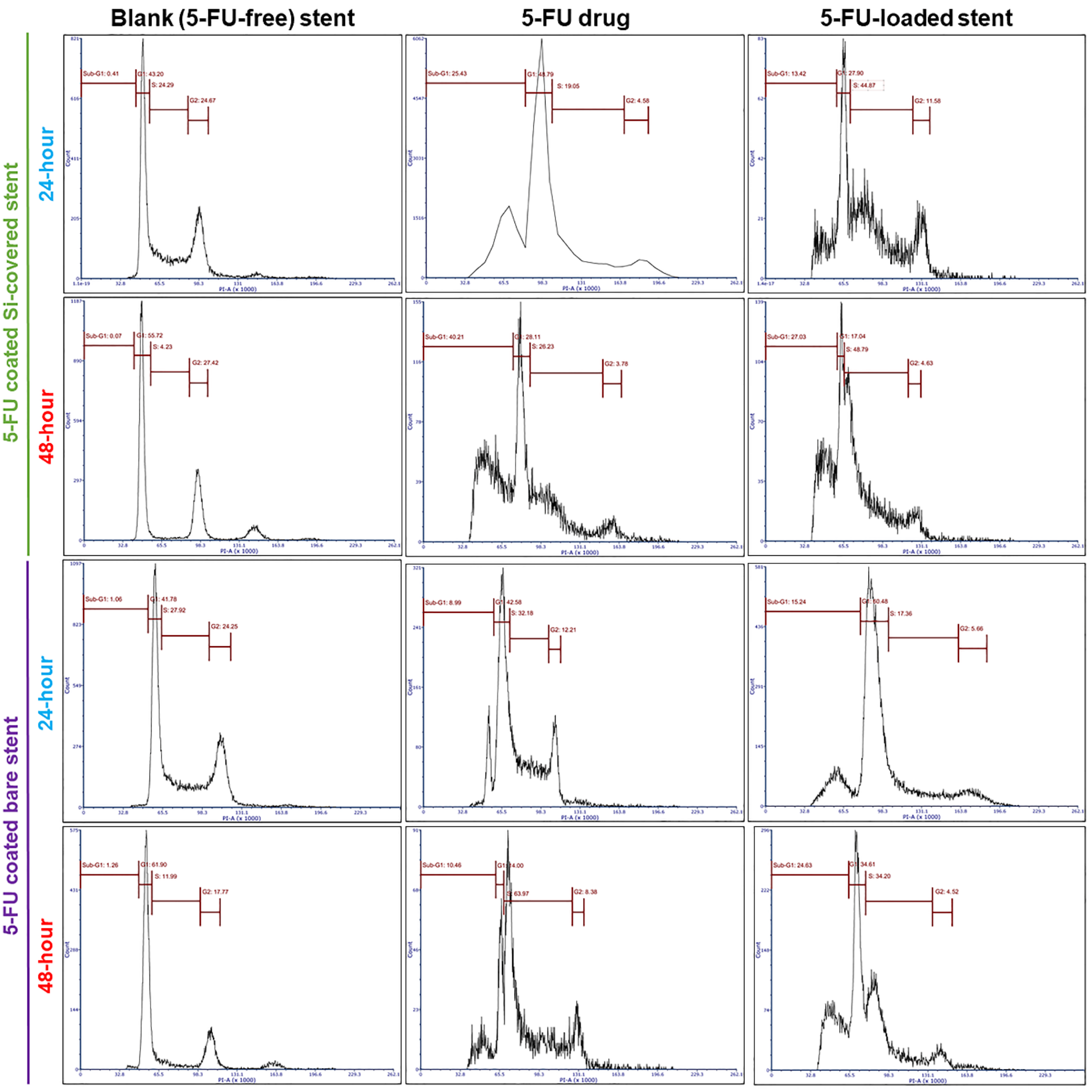

Figure S13. Cell cycle distribution (%) of HCT-116 human colon cancer cells in different treatment groups at 24 and 48 h.

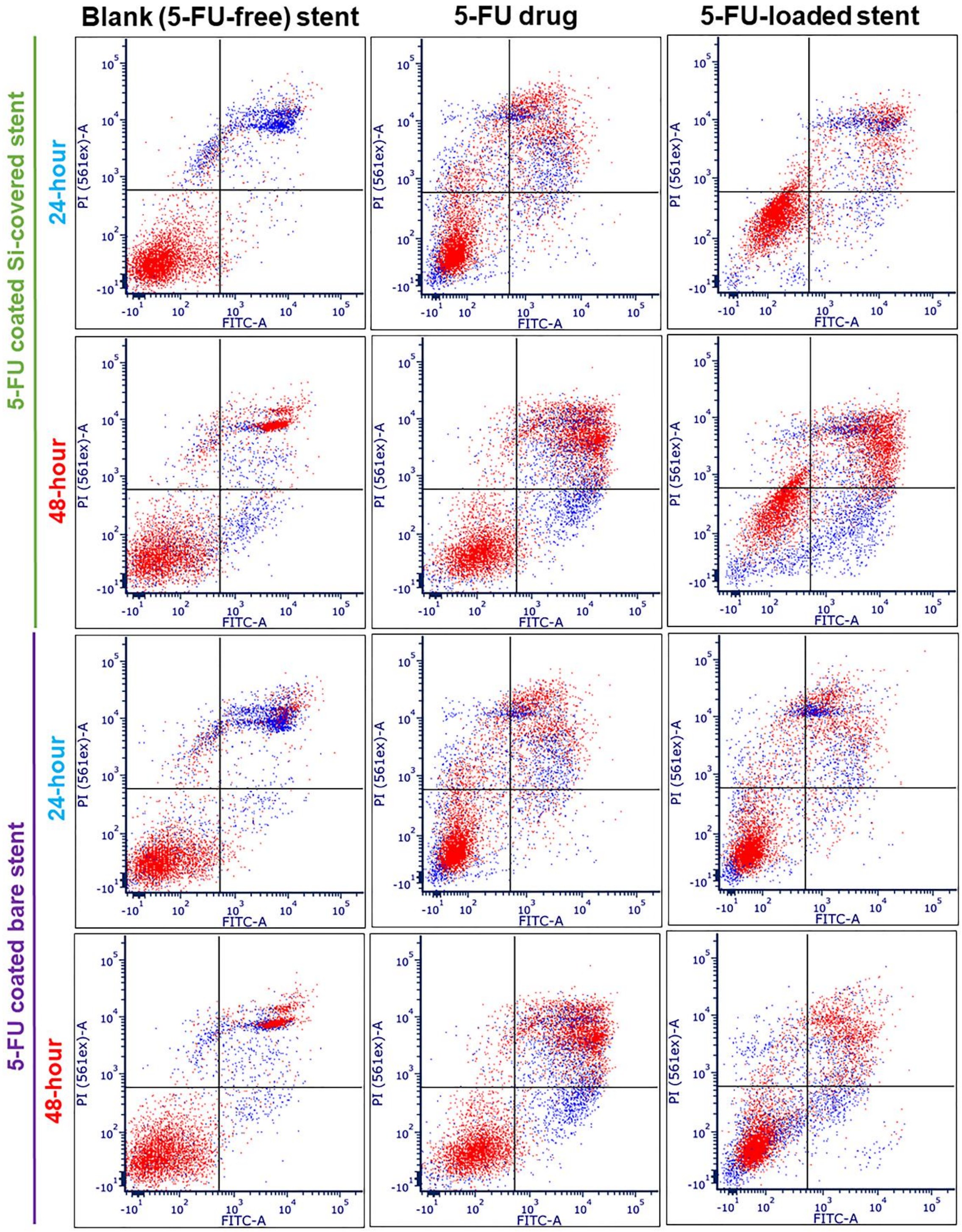

Figure S14. Apoptosis progression in HCT-116 human colon cancer cells after treatment with drug-free **Si-PU-PEVA** and **B-PU-PEVA** stents, 5FU drug (positive control) and drug-loaded **Si-PU$_{5FU}$-PEVA** and **B-PU$_{5FU}$-PEVA** stents for 24 and 48 h, as analysed by a flow cytometry-based annexin V-FITC/propidium iodide (PI) assay. Quadrants upper left, upper right, lower left and lower right represent necrotic, late apoptotic, viable and early apoptotic cells, respectively.

Table S1. Composition of the stent coating solutions.

Layers	Component	Concentration			
		in liquid medium		on dry basis	
Drug-loaded base layer	Polyurethane (PU)	17.50	% w/v	93.5	% w/w
	Tetrahydrofuran (THF)	86.30	% v/v	--	
	5-Fluorouracil (5FU)	1.22	% w/v	6.5	% w/w
	N,N-Dimethylformamide (DMF)	13.70	% v/v	--	
Drug-free top layer	Poly(ethylene-co-vinyl acetate) (PEVA)	26	% w/v	100	% w/w
	Dichloromethane (DCM)	100	% v/v	--	

Table S2. Optimised parameters used for dip-coating the commercial nitinol stents.

Coating layers	Parameters	Values	
		Si membrane-covered nitinol stents	Bare nitinol stents
5FU-loaded PU basecoat	Immersion angle*	90°	90°
	Dwell time#	Not more than 2 s	Not more than 2 s
	Withdrawal speed	1.07 ± 0.05 mm/s@	1.07 ± 0.11 mm/s@
	Number of dipping-withdrawal cycles	1	1
Drug-free PEVA topcoat	Immersion angle*	90°	90°
	Dwell time#	Not more than 2 s	Not more than 2 s
	Withdrawal speed	1.43 ± 0.06 mm/s@	1.56 ± 0.08 mm/s@
	Number of dipping-withdrawal cycles	1	1

Si, Silicone; PU, Polyurethane; PEVA, Poly(ethylene-co-vinyl acetate); *With respect to the surface of coating solution; #Stent retention time in the respective coating solution; @Data are expressed as mean (n = 10) ± SD

Table 3. Description/Type of samples used in different characterisation studies of the films and coated stents.

Characterisation tests	Sample description/details
Photoacoustic Fourier-transform infrared (PA FT-IR) spectroscopy	Silicon wafer substrates dip-coated with different coating layers (PU-5FU base layer or PEVA top-coated 5FU-PU layer or only PEVA layer)
X-ray diffraction (XRD), differential scanning calorimetry (DSC), and thermogravimetric analysis (TGA)	5FU-loaded PU and drug-free (blank) PU solvent cast films
X-ray photoelectron spectroscopy (XPS)	Single-layered (only basecoat) and bilayered (both basecoat and topcoat) dip-coated films on glass microscope slides
Scanning electron microscopy (SEM)	5FU-loaded PU base layer and PU-5FU-loaded PEVA top layer coated nitinol stent cut pieces
In vitro drug release in RPMI-1640 cell culture medium supplemented with 10% (v/v) FBS	5FU-loaded bilayer coated Si-covered and bare nitinol stent cut pieces of approx. equal weights

PU, Polyurethane; PEVA, Poly(ethylene-co-vinyl acetate); RPMI, Roswell Park Memorial Institute; FBS, Fetal bovine serum; Si, Silicone

Table S4. XRD data of pure 5FU drug and 6.5 % w/w 5FU-loaded PU monolayer film.

Sample	Measured ~ 2θ (°)	Peak intensity (counts)	*d*-spacing (Å)*
5FU drug	28.6	481469.1	3.123
5FU drug	37.8	32825.4	2.381
5FU (6.5 % w/w)-polyurethane (PU) film	28.1	24679.5	3.182
5FU (6.5 % w/w)-PU film	37.7	30724.3	2.386

*d-Spacing values were calculated using the Bragg's equation: $\lambda = 2(d\text{-spacing}) \sin \theta$; where λ is the wavelength of X-ray, 2θ is the X-ray scattering angle.
